# Targeting ANXA1/TRKA axis enhances immunotherapy sensitivity in neural invasion-positive gastric cancer

**DOI:** 10.1186/s43556-026-00444-1

**Published:** 2026-04-09

**Authors:** Tianlu Jiang, Peng Zhou, Yikai Shen, Jie Lin, Ying Li, Xusheng Shen, Lang Fang, Penghui Xu, Zekuan Xu, Linjun Wang, Yiwen Xia

**Affiliations:** 1 Department of General Surgery, The Affiliated Wuxi People’s Hospital of Nanjing Medical University, Wuxi People’s Hospital, Wuxi Medical Center, Nanjing Medical University, Wuxi, Jiangsu Province China; 2 Gastric Cancer Center, The First Affiliated Hospital of Nanjing Medical University, Nanjing, Jiangsu Province China; 3 Department of General Surgery, The First Affiliated Hospital of Nanjing Medical University, Nanjing, Jiangsu Province China; 4 Department of General Surgery, Jiangnan University Medical Center, Wuxi, Jiangsu Province, China; 5 Department of General Surgery, Hepatobiliary Surgery, Huashan Hospital & Cancer Metastasis Institute, Fudan University, Shanghai, China; 6 Institute for Gastric Cancer Research, Nanjing Medical University, Nanjing, Jiangsu Province China; 7 Jiangsu Key Lab of Cancer Biomarkers, Prevention and Treatment, Collaborative Innovation Center for Personalized Cancer Medicine, Nanjing Medical University, Nanjing, Jiangsu Province China

**Keywords:** Gastric cancer, Neural invasion, T cell exhaustion, ANXA1, TRKA, Immune evasion

## Abstract

Gastric cancer (GC) remains a leading cause of cancer-related morbidity and mortality worldwide. Neural invasion (NI) is a common pathological behavior that worsens the prognosis in GC. However, the immune microenvironment of neural invasion-positive GC (NI^+^GC) and its potential therapeutic implications remain poorly defined. Here, we performed single-cell RNA sequencing (scRNA-seq) on tumor specimens from patients with NI^+^GC and neural invasion-negative GC (NI^−^GC) to comprehensively delineate the immune landscape. Our analysis revealed a significant enrichment of exhausted ANXA1^+^CD8^+^T cells within NI^+^GC tissues, which was validated by flow cytometry and multiplex immunohistochemistry assays. Clinically, patients with greater infiltration of ANXA1^+^CD8^+^ T cells had worse overall survival and disease progression. Mechanistically, ANXA1 bound to TRKA via N-terminal region, blocking NEDD4L-mediated ubiquitination and degradation of TRKA, thereby suppressing glycolytic metabolism and driving CD8⁺T cell exhaustion. Tumor-derived nerve growth factor (NGF) further amplified this exhaustion via TRKA engagement. Importantly, treatment with an ANXA1-derived peptide (A11) combined with TRKA inhibitors synergistically reversed T cell exhaustion and suppressed tumor growth in preclinical models. These findings unveil distinctive features of the immune microenvironment in NI^+^GC and elucidate the underlying molecular mechanisms of ANXA1/TRKA axis in facilitating immune evasion, which offer new insights for enhancing the sensitivity of immunotherapy to NI^+^GC patients.

**Supplementary Information:**

The online version contains supplementary material available at 10.1186/s43556-026-00444-1.

## Introduction

Gastric cancer (GC), one of the most prevalent malignancies globally, ranks fifth in both incidence and mortality among malignant tumors [1]. China has a significant GC burden, accounting for approximately 42.6% of global GC cases and 45.0% of related deaths, imposing a severe health challenge [2]. At present, the treatment methods for GC mainly include endoscopic treatment, surgical treatment, and chemotherapy. Although these treatment options have achieved certain therapeutic effects, some cases of GC recurrence, drug resistance, and distant metastasis still occur in patients with advanced GC, leading to poor quality of life and limited survival [3]. In recent years, research on immunotherapy for GC has been continuous, and significant progress has been made. The combination of immunotherapy with chemotherapy, targeted drugs, or other immunomodulatory drugs, has gradually become a research hotspot [4]. However, only a minority of GC patients benefit from immunotherapy, likely because of the high heterogeneity and complexity of the tumor microenvironment [5]. Therefore, understanding the molecular heterogeneity within GC is crucial for improving the effectiveness of immunotherapies.

Neural invasion (NI) refers to the pathological process in which tumor cells infiltrate, surround and spread along nerves, causing neuropathy, accompanied by symptoms such as pain, numbness, and functional impairment, which are common in cancers of the pancreas, prostate, stomach, and colorectum [6–8]. Previous studies have shown that neural invasion is important for evaluating disease progression and prognosis in GC patients, and is also directly associated with the risk of early recurrence after surgical treatment [9, 10]. The retrospective analysis from our center indicated that GC patients with NI had worse outcomes than those without NI [11].

The tumor microenvironment (TME) is a complex ecosystem surrounding tumor cells that comprises not only cancer cells but also immune cells, stromal cells, vascular systems, metabolites, and signaling molecules [12]. T-cell subpopulations with different functional states play vastly different roles in the occurrence and development of tumors and are important contributors to differences in patient survival and prognosis [13]. Notably, exhausted T cells actively participate in the development of an immunosuppressive tumor microenvironment through various mechanisms, including high expression of inhibitory receptors and the release of inhibitory cytokines, greatly limiting the effectiveness of antitumor immunotherapy [14]. Uncovering the unique immunological features of the microenvironment in NI^+^GC is pivotal for devising more specific treatment strategies and improving therapeutic outcomes for this particular subtype. The advent and evolution of scRNA-seq have enabled researchers to analyze gene expression at the individual cell level, which is crucial for revealing cellular molecular diversity and identifying rare cell types [15, 16]. Numerous studies have constructed single-cell atlases of GC microenvironments, elucidating cellular heterogeneity and intercellular interactions during the occurrence and development of GC [17–19]. However, no study has specifically targeted NI^+^GC to clarify the cellular populations and intrinsic molecular characteristics of this microenvironment.

Annexin A1 (ANXA1), a member of the phospholipid-binding protein family, is involved in malignant biological behaviors such as proliferation, metastasis, and drug resistance [20]. ANXA1 also exerts immunosuppressive effects by promoting M2 macrophages polarization, modulating the Th1/Th2 cell ratio, and inducing Treg infiltration in the tumor immune microenvironment [21]. In GC, elevated ANXA1 expression is associated with peritoneal metastasis and a poor prognosis [22]. Nonetheless, the role of ANXA1 in CD8^+^ T cells and the function of ANXA1^+^CD8^+^T cells remain unexplored in GC.

In this study, we employed scRNA-seq coupled with gene expression profiling, cell type identification, and intercellular signaling pathway analysis to reveal the differences in cell subtypes and characteristics between NI^+^GC and NI^−^GC. Upregulation of ANXA1^+^CD8^+^T cells was identified in NI^+^GC. A series of experiments involving flow cytometry, coculture assays and multiplex immunofluorescence were conducted to elucidate the role and intrinsic molecular mechanisms of ANXA1^+^CD8^+^T cells in promoting immune escape in NI^+^GC. Our findings shed light on the underlying molecular mechanisms and offer new perspectives for enhancing the sensitivity of immunotherapy in NI^+^GC patients.

## Results

### Differences in the tumor microenvironment between NI^+^GC and NI^−^GC

To clarify the characteristic immune microenvironment of NI^+^GC, we collected fresh tumor samples from GC patients who underwent gastrectomy to conduct scRNA-seq. Among them, 3 patients had postoperative pathological confirmation of negative neural invasion (marked as GC1, GC3, and GC5), and 3 patients were positive neural invasion (marked as GC2, GC4, and GC6) (Supplementary Table 1).

**Fig. 1:**
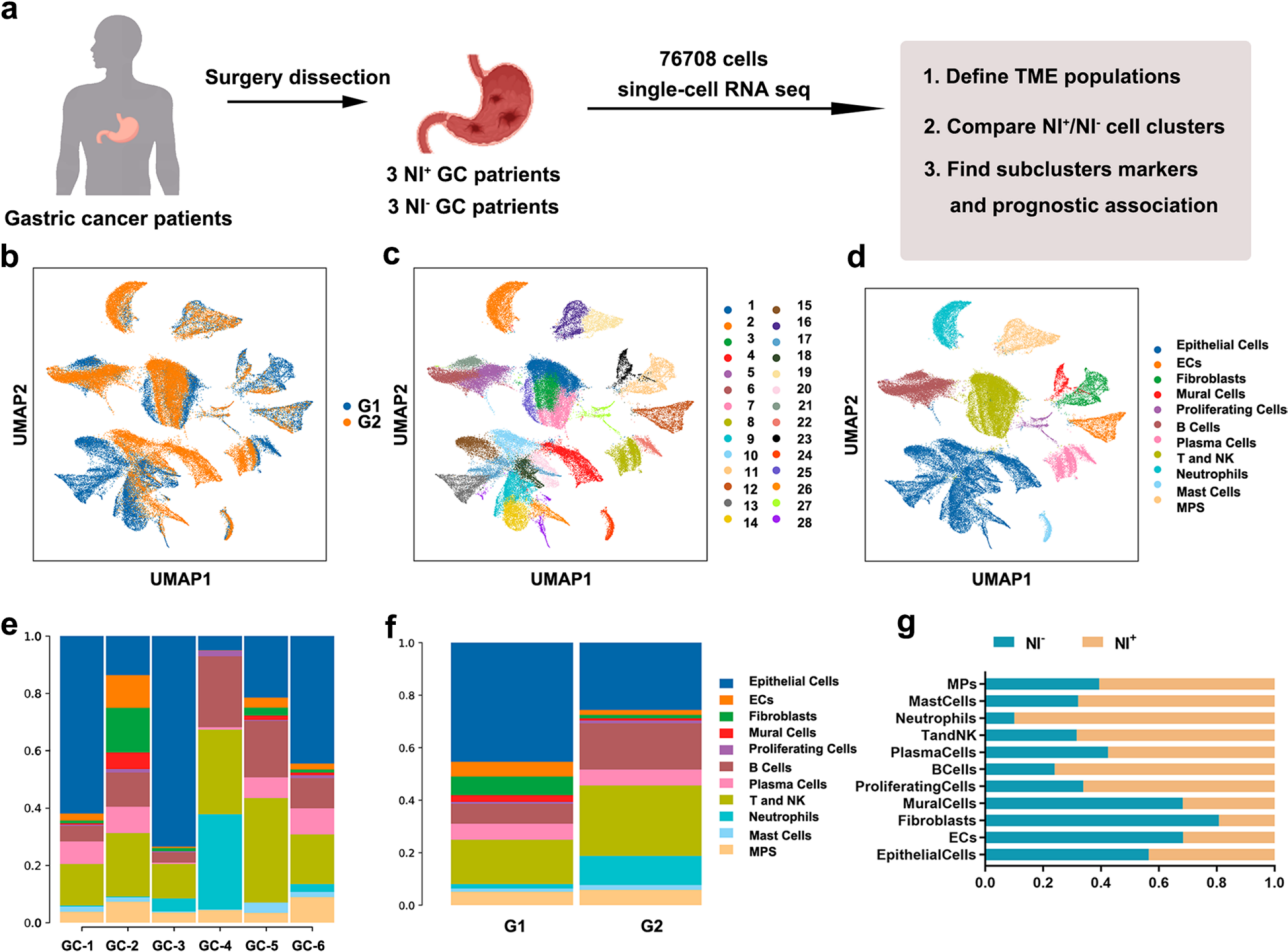
Differences in tumor microenvironment between NI^+^GC and NI^−^GC. **a** Summary of the workflow depicting sample processing, single-cell RNA sequencing (scRNA-seq) and subsequent analytical processes; **b** Uniform Manifold Approximation and Projection (UMAP) plot of the 76,708 analyzed single cells from 6 patients, colored by cellular tissue origin; Each dot in the UMAP represents a single cell; **c** UMAP plot based on cell clusters, colored by cell subclusters; **d** UMAP plot based on cell type, colored by major cell types; **e** Fractions of cell types detected in each sample, colored as in Fig. 1d. **f** Fractions of cell types detected in each group, colored as in Fig. 1d; **g** The distribution of cells derived from different sample origins. G1: neural invasion-negative gastric cancer (NI^−^GC), G2: neural invasion-positive gastric cancer (NI.^+^GC)

Fresh tumor samples were quickly digested into single-cell suspensions for scRNA-seq (Fig. 1a). After quality control, 76,708 cells with more than 200 detectable genes were obtained, 57.7% of which were from NI^+^GC samples and 42.3% were from NI^−^GC samples. (Fig. 1b). We performed unsupervised dimensionality reduction clustering on cells to identify cell clusters on the basis of their expression patterns. After normalization and principal component analysis of the read counts, we obtained a total of 28 cell clusters (Fig. 1c). We then classified them into 11 main cell types based on the expression of classic marker genes and significantly differentially expressed genes (Fig. 1d) [23], including epithelial cells, endothelial cells, fibroblasts, mural cells, proliferating cells, B cells, plasma cells, T and NK cells, neutrophils, mast cells, and mononuclear phagocytes (MPs) (Fig. S1). In different samples and groups (Fig. 1e-f), the proportions of major cell types varied greatly, indicating significant tumor heterogeneity. Compared with those in the NI^−^GC samples, the proportions of monocytes, mast cells, neutrophils, T and NK cells, plasma cells, B cells and proliferating cells were greater in the NI^+^GC samples (Fig. 1g).

### CD8^+^T cells in NI^+^GC were in a functionally exhausted state

Tumor-infiltrating CD8^+^T cells are primary cytotoxic immune cells, with activated CD8^+^T cells exerting their killing effect on tumor cells through the secretion of perforin, granzymes, and cytokines such as TNF-α and IFN-γ, thereby inhibiting tumor progression [24]. Multiple studies have confirmed that the functional status of CD8^+^T cells is significantly correlated with tumor malignancy and patient prognosis [25, 26]. UCell scores revealed a significantly reduced cytotoxic ability of T and NK cell in NI^+^GC, preliminarily suggesting that CD8^+^T cells in NI^+^GC were in an exhausted state (Fig. S2a).

Under chronic antigen exposure in tumors or during persistent infections, T cells exhibit features of upregulated immune checkpoint expression, diminished cytokine secretion, and a reduced capacity to kill tumor cells, a state of functional impairment known as T-cell exhaustion [27]. T-cell exhaustion is intricately linked with tumor progression and represents immune dysfunction in cancer patients, significantly impacting the efficacy of immunotherapeutic approaches [28]. We further investigated the functional status of CD8^+^T cells in NI^+^GC and NI^−^GC.

**Fig. 2:**
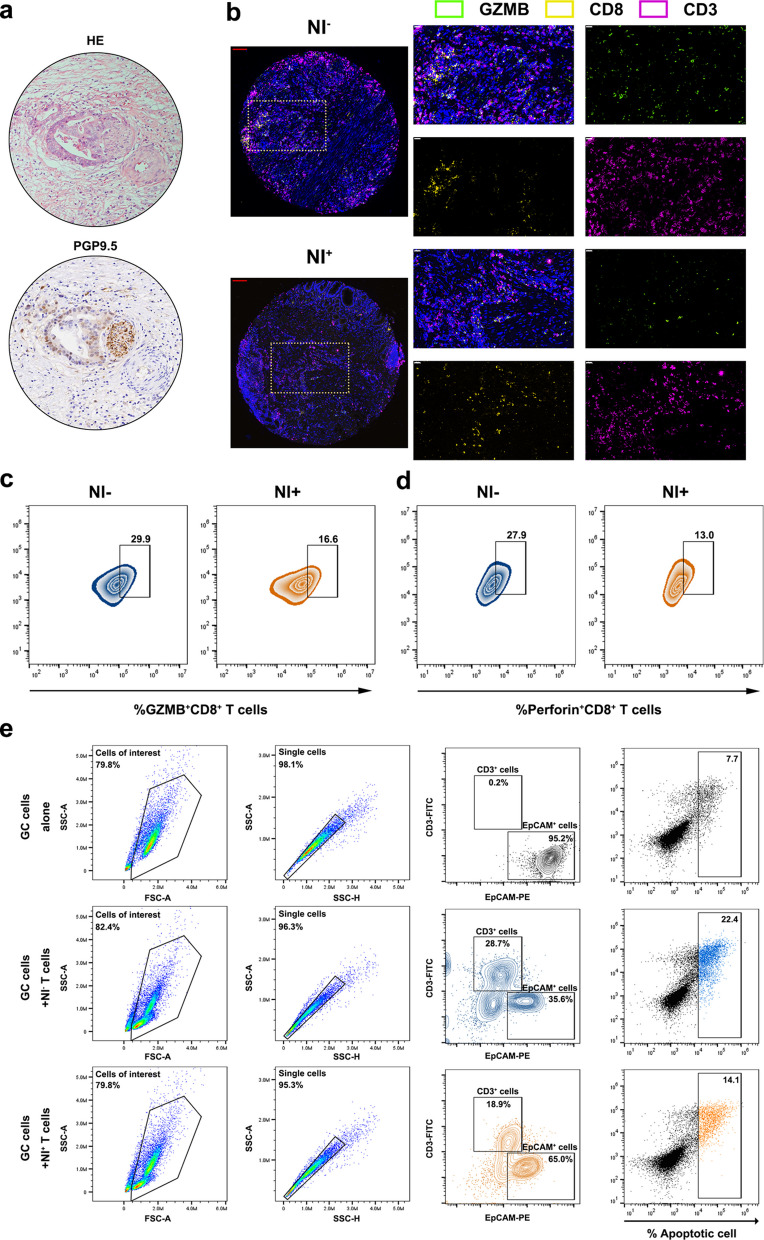
The functional status of CD8^+^T cells in NI^+^GC and NI^−^GC. **a** Pathological identification of NI^+^GC: Upper: Hematoxylin–eosin staining(H&E) showed tumor cells growing along the nerve fibers, with destruction of the perineurium and associated infiltration by tumor cells; Lower: Immunohistochemistry staining for PGP9.5 confirmed the presence of neural invasion in gastric cancer; **b** Representative images of tissue microarrays of NI^−^GC and NI^+^GC, multiplex immunohistochemistry (mIHC) with anti-GZMB, anti-CD8 and anti-CD3, red scale bar: 100 μm, white scale bar: 20 μm; *n* = 30; **c-d** Flow cytometry of GZMB^+^CD8^+^T cells and Perforin^+^CD8^+^T cells in NI^−^GC and NI^+^GC; **e** The apoptosis rate of AGS separately co-cultured with CD8^+^T cells derived from NI^−^GC and NI.^+^GC patients, *n* = 10

First, we conducted multiplex immunohistochemistry on the constructed GC tissue microarrays, which revealed a significantly reduced proportion of GZMB^+^CD8^+^T cells in NI^+^GC group (Fig. 2a-b, Fig. S2b). CD8^+^T cells were subsequently isolated from the peripheral blood of NI^+^GC and NI^−^GC patients, after which the cells were cultured in vitro and assessed by flow cytometry (Fig. S2c). The results indicated that CD8^+^T cells derived from NI^+^GC patients expressed lower perforin and granzymes (Fig. 2c-d, Fig. S2d-e) and secreted fewer effector cytokines, such as IFN-γ and TNF-α (Fig. S2f). When GC cells were separately cocultured with CD8^+^ T cells derived from the NI^+^GC and NI^−^GC samples, the apoptosis rate was significantly lower in the former group (Fig. 2e, Fig. S2g). These results indicated that the CD8^+^T cells derived from NI^+^GC patients exhibited significantly impaired cytotoxic function and were in a state of functional exhaustion.

### The infiltration of ANXA1^+^CD8^+^T cells was elevated in NI^+^GC

**Fig. 3:**
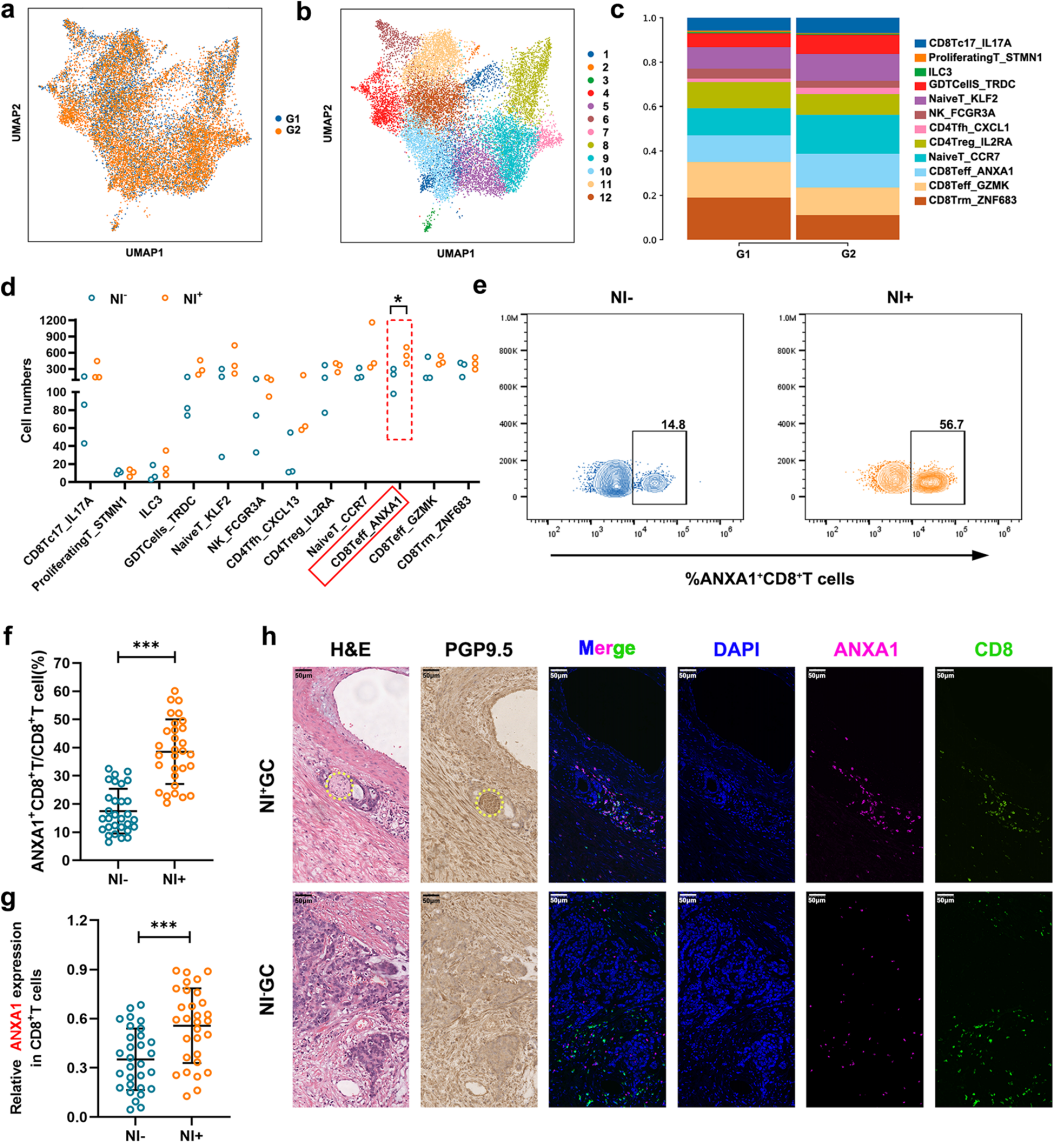
The differential infiltration of CD8^+^T cell subsets between NI^+^GC and NI^−^GC. **a** UMAP plot of the T and NK cells, colored by cellular tissue origin; **b** UMAP plot based on T and NK cell clusters, clusters are labeled with inferred cell types, each color represents one cluster; **c** Fractions of cell clusters detected in each group, colored as in Fig. 3b; **d** The cell number of the indicated T and NK cell clusters within different GC samples from scRNA-seq; **e–f** The proportion of ANXA1^+^CD8^+^T cells in NI^+^GC and NI^−^GC samples (*n* = 30); **g** The mRNA levels of ANXA1 in CD8^+^T cells of NI^+^GC and NI^−^GC samples (*n* = 30); **h** MIHC of ANXA1^+^CD8^+^T cells in NI^+^GC and NI^−^GC samples with matching H&E staining and PGP9.5 immunohistochemical staining; yellow cycle: nerve, scale bar:50 μm. G1: NI^−^GC, G2:NI^+^GC;The data are presented as the means ± SD. *p*-values were determined by two-tailed unpaired Student’s t-test. *p < 0.05, **p < 0.01, and ***p < 0.001 versus the control group

Distinct CD8^+^T cell subsets exhibit wide-ranging variations in morphology, surface marker expression, functional potential, and metabolic characteristics. These different subtypes are intimately associated with variations in treatment responsiveness and prognosis among patients [29]. To investigate the differential infiltration of CD8^+^T cell subsets between NI^+^GC and NI^−^GC, 15,830 T and NK cells were subjected to unsupervised clustering analysis, reannotation, and identification, yielding 12 distinct cellular subclusters annotated as CD8Tc17_IL17A, ProliferatingT_STMN1, Group 3 innate lymphoid cells (ILC3), GDTCells_TRDC, NaiveT_KLF2, NK_FCGR3A, CD4Tfh_CXCL13, CD4Treg _IL2RA, NaiveT_CCR7, CD8Teff_ANXA1, CD8Teff_GZMK, and CD8Trm_ZNF683 (Fig. 3a-b, Fig. S3a).

Despite considerable variations in infiltration levels, all 12 T and NK cell subclusters were identified in both NI^+^GC and NI^−^GC samples (Fig. 3c). Notably, the infiltration of CD8Teff_ANXA1 subcluster was significantly increased in NI^+^GC (*p* = 0.0287, herein referred to as ANXA1^+^CD8^+^T cells) (Fig. 3d, Fig. S3b-c). Flow cytometry showed that the proportion of ANXA1^+^CD8^+^T cells was significantly increased in NI^+^GC patients (Fig. 3e-f). Quantitative real-time PCR assays also revealed marked upregulation of ANXA1 mRNA levels in CD8^+^ T cells derived from NI^+^GC samples (Fig. 3g). Consistent with these findings, multiplex immunohistochemistry of GC tissues further substantiated this observation, demonstrating elevated ANXA1^+^CD8^+^T cells in NI^+^GC samples (Fig. 3h, Fig. S3d). We then scored the ANXA1^+^CD8^+^T cell signature in GC tissues and confirmed that greater infiltration of ANXA1^+^CD8^+^T cells was associated with a poorer prognosis in GC patients (Fig. S3e-f).

### ANXA1^+^CD8^+^T cells were exhausted in the NI^+^GC group

**Fig. 4:**
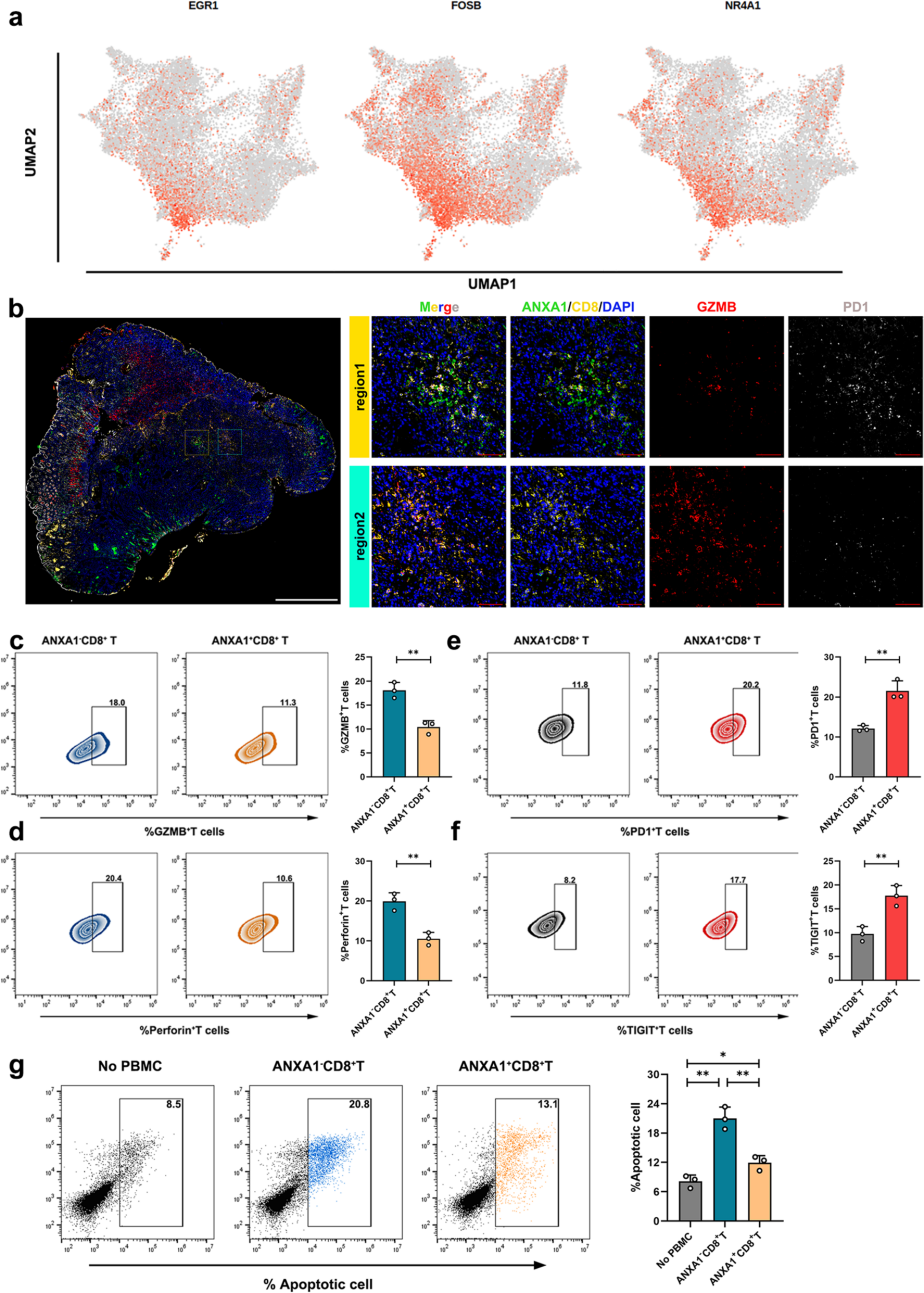
Functional characteristics of the upregulated ANXA1^+^CD8^+^T cells in NI^+^GC. **a** UMAP plot color-coded (gray to orange) to represent the expression levels of the marker genes: EGR1, FOSB, NR4A1 in ANXA1^+^CD8^+^T cells; **b** MIHC of ANXA1^+^CD8^+^T cells in GC samples with anti-ANXA1(green), anti-CD8(yellow), anti-GZMB (red) and anti-PD1(gray), white scale bar:50 mm, red scale bar:80 μm; **c-d** The positive proportion of effector factors (GZMB、Perforin) in ANXA1^−^CD8^+^T cells and ANXA1^+^CD8^+^T cells; **e–f** The positive proportion of exhausted factors (PD1、TIGIT) in ANXA1^−^CD8^+^T and ANXA1^+^CD8^+^T cells; **g** The apoptosis rate of cancer cells co-cultured with ANXA1^−^CD8^+^T cells and ANXA1^+^CD8^+^T cell. GC cells, ANXA1^−^CD8^+^T cells and ANXA1^+^CD8^+^T cell were harvested from the same NI^+^GC patient. All experiments were repeated 3 times with consistent results. The data are presented as the means ± SD. *p*-values were determined by two-tailed unpaired Student’s t-test. *p < 0.05, **p < 0.01, and ***p < 0.001 versus the control group

We subsequently investigated the function of ANXA1^+^CD8^+^T cells in GC. The upregulated genes in ANXA1^+^CD8^+^T cells, including EGR1, FOSB, and NR4A1, function as transcriptional regulators of proliferation, the DNA damage response, and apoptosis through the modulation of target gene transcription (Fig. 4a). Notably, NR4A1 is pivotal for inducing T effector cell dysfunction and fostering an immunosuppressive microenvironment [30, 31], suggesting that ANXA1^+^CD8^+^T cells are associated with immune suppression.

Multiplex immunohistochemistry revealed that regions with high infiltration of ANXA1^+^CD8^+^T cells exhibited elevated PD1 expression and reduced GZMB expression, whereas regions with low infiltration presented the opposite results (Fig. 4b, Fig. S3g-h). To further validate the exhausted state of ANXA1^+^CD8^+^T cells, we isolated ANXA1^+^CD8^+^T cells and ANXA1^−^CD8^+^T cells from NI^+^GC samples. The expression of effector molecules (GZMB and perforin) was significantly lower in ANXA1^+^CD8^+^T cells than in ANXA1^−^CD8^+^T cells (Fig. 4c-d), whereas the opposite pattern was observed for exhaustion markers (PD1 and TIGIT) (Fig. 4e-f). Also, the apoptosis level of cancer cells cocultured with ANXA1^+^CD8^+^T cells was significantly lower than that of cancer cells cocultured with ANXA1^−^CD8^+^T cells (Fig. 4g). These findings confirmed a notably increased infiltration of exhausted ANXA1^−^CD8^+^T cells in the NI^+^GC group.

### ANXA1^+^CD8^+^T cells exhibited diminished glycolytic activity

Thus far, we have preliminarily shown that the upregulated ANXA1^+^CD8^+^ T cells in NI^+^GC were in a state of functional exhaustion. However, the intrinsic mechanisms underlying their exhaustion remained unclear. We first conducted pathway enrichment analysis in ANXA1^+^CD8^+^T cells and ANXA1^−^CD8^+^T cells. The upregulated genes in ANXA1^+^CD8^+^T cells are predominantly involved in processes such as ubiquitin–proteasome degradation, IL-17 signaling pathways, and Th17 cell differentiation (Fig. S4a). Conversely, genes downregulated in ANXA1^+^CD8^+^ T cells are associated primarily with T-cell receptor signaling pathways, chemokine responses, and other activation pathways crucial for T-cell function. Notably, we also observed the glycolysis/gluconeogenesis metabolic pathway was downregulated in ANXA1^+^CD8^+^ T cells (Fig. S4b). Further gene set variation analysis (GSVA) for metabolic pathway enrichment revealed that ANXA1^+^CD8^+^T cells negatively regulated glucose metabolism related pathways, including glycolysis/gluconeogenesis, glycogen synthesis, and the tricarboxylic acid (TCA) cycle (Fig. S4c).

Extensive studies have confirmed that glucose metabolism constitutes a predominant energy metabolism pathway following T-cell activation, and plays an indispensable role in T-cell proliferation and functional execution. The metabolic reprogramming of glucose metabolism in T cells is intimately linked to alterations in their functional states [27, 32, 33]. We then individually examined the mRNA and protein expression levels of key glycolytic genes, which indicated a significantly lower expression in ANXA1^+^CD8^+^T cells (Fig. S4d-e). Extracellular acidification rate (ECAR) and oxygen consumption rate (OCR) assays also confirmed that ANXA1^+^CD8^+^T cells exhibited reduced metabolic activity compared with that of ANXA1^−^CD8^+^T cells (Fig. S4f-i). These results suggested that aberrant glucose metabolism in ANXA1^+^CD8^+^T cells was a pivotal factor underlying their functional exhaustion.

### ANXA1 competed with NEDD4L for binding to TRKA and inhibited its degradation via the ubiquitin–proteasome pathway

**Fig. 5:**
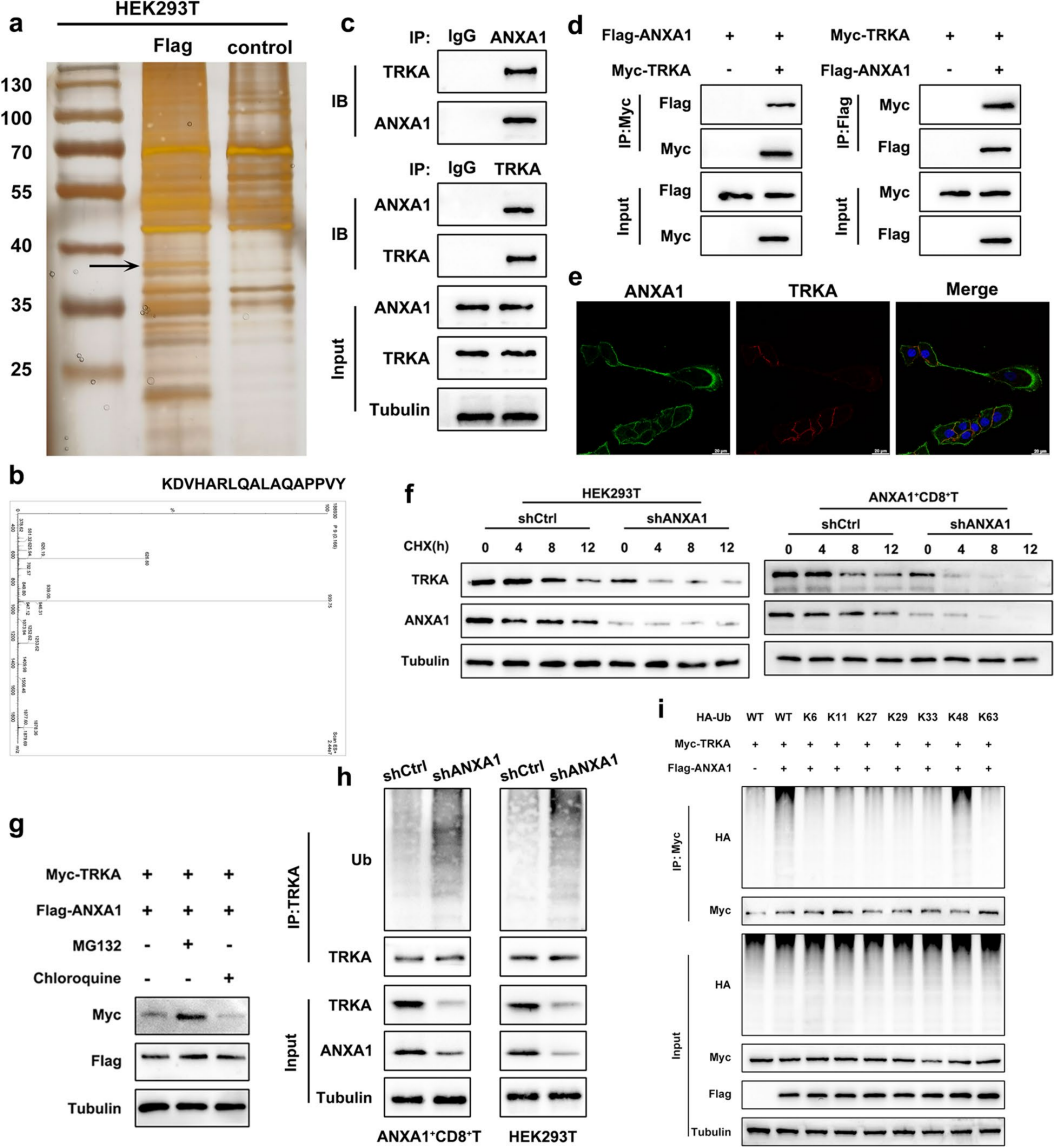
ANXA1 bound to TRKA and inhibited its degradation via the ubiquitin–proteasome pathway. **a** Immunoprecipitation and silver staining of HEK293T cells stably expressing Flag-ANXA1; **b** Mass spectrum of TRKA; **c-d** Co-immunoprecipitation (Co-IP) was conducted to verify the binding of ANXA1 to TRKA in ANXA1^+^CD8^+^T cells and HEK293T cells; **e** Representative confocal immunofluorescence microscopy image of ANXA1^+^CD8^+^T cells staining for ANXA1 (green) and TRKA (red) and counterstained with DAPI. Scale bars:20 μm; **f** TRKA protein expression in HEK293T cells and ANXA1^+^CD8^+^T cells after treated with cycloheximide (CHX) of the indicated time; **g** TRKA protein expression after MG132(10 μm,24 h)/chloroquine (50 μm,24 h) treatment; **h** Co-IP of TRKA ubiquitination in ANXA1^+^CD8^+^T cells and HEK293T cells after interfering with ANXA1; **i** Ubiquitin binding sites of TRKA. All experiments were repeated 3 times with consistent results

We subsequently explored the molecular mechanism through which ANXA1 regulated glucose metabolism in CD8^+^T cells. Through co-immunoprecipitation (co-IP) followed by mass spectrometry, we focused on the tyrosine kinase receptor TRKA (Fig. 5a-b). Given that TRKA has been reported to regulate cell differentiation by affecting glycolytic activity, and ANXA1 can influence the biological behavior of malignant tumors by binding to and modulating the expression or activity of tyrosine kinase receptors such as insulin receptor and EGFR [34, 35], we hypothesized that TRKA could act as a potential downstream target through which ANXA1 regulates glucose metabolism in CD8^+^T cells. Co-IP and immunofluorescence confirmed the interaction between ANXA1 and TRKA in ANXA1^+^CD8^+^T cells (Fig. 5c-e). Upon the knockdown of TRKA in ANXA1^+^CD8^+^T cells, we observed a significant increase in the glycolytic activity (Fig. S5a-b) and elevated expression of key glycolysis-related genes (Fig. S5c), while the expression of exhaustion markers was markedly reduced (Fig. S5d-e). Interfering with TRKA also reversed the suppressive effect on glucose metabolism and T-cell exhaustion induced by ANXA1 overexpression. Collectively, these findings showed that ANXA1, by binding to TRKA, inhibited glucose metabolism and thereby induced exhaustion of ANXA1^+^CD8^+^T cells.

We next investigated whether ANXA1 regulated the expression of TRKA. A significant reduction in TRKA protein expression was observed when ANXA1 was knocked down (Fig. S5f), while no substantial difference was detected at the mRNA level (Fig. S5g). Cycloheximide (CHX) assay was conducted to assess the impact of ANXA1 on TRKA protein stability. As depicted in Fig. 5f and Fig. S5h-I, TRKA was degraded faster when ANXA1 was knocked down, suggesting that ANXA1 effectively increased the protein stability of TRKA. Treatment with the proteasome inhibitor MG132, rather than chloroquine, effectively reversed the decrease in TRKA levels, indicating that ANXA1 modulated TRKA protein expression via the proteasomal pathway (Fig. 5g). When ANXA1 was knocked down, a significant elevation of TRKA ubiquitination was observed (Fig. 5h), suggesting that ANXA1 enhanced TRKA protein stability by inhibiting its ubiquitin-mediated degradation. To further screen the type of ubiquitin involved in the ANXA1-mediated ubiquitination of TRKA, we transfected HEK293T cells with various mutant forms of ubiquitin and assessed the ubiquitination levels after 12 h of treatment with MG132. The regulation of TRKA ubiquitination by ANXA1 was detected only in the ubiquitin mutants in which the K48 site was retained (Fig. 5i). These findings suggested that ANXA1 bound to TRKA and inhibited its degradation via the ubiquitin–proteasome pathway, thereby increasing the protein stability of TRKA.

**Fig. 6:**
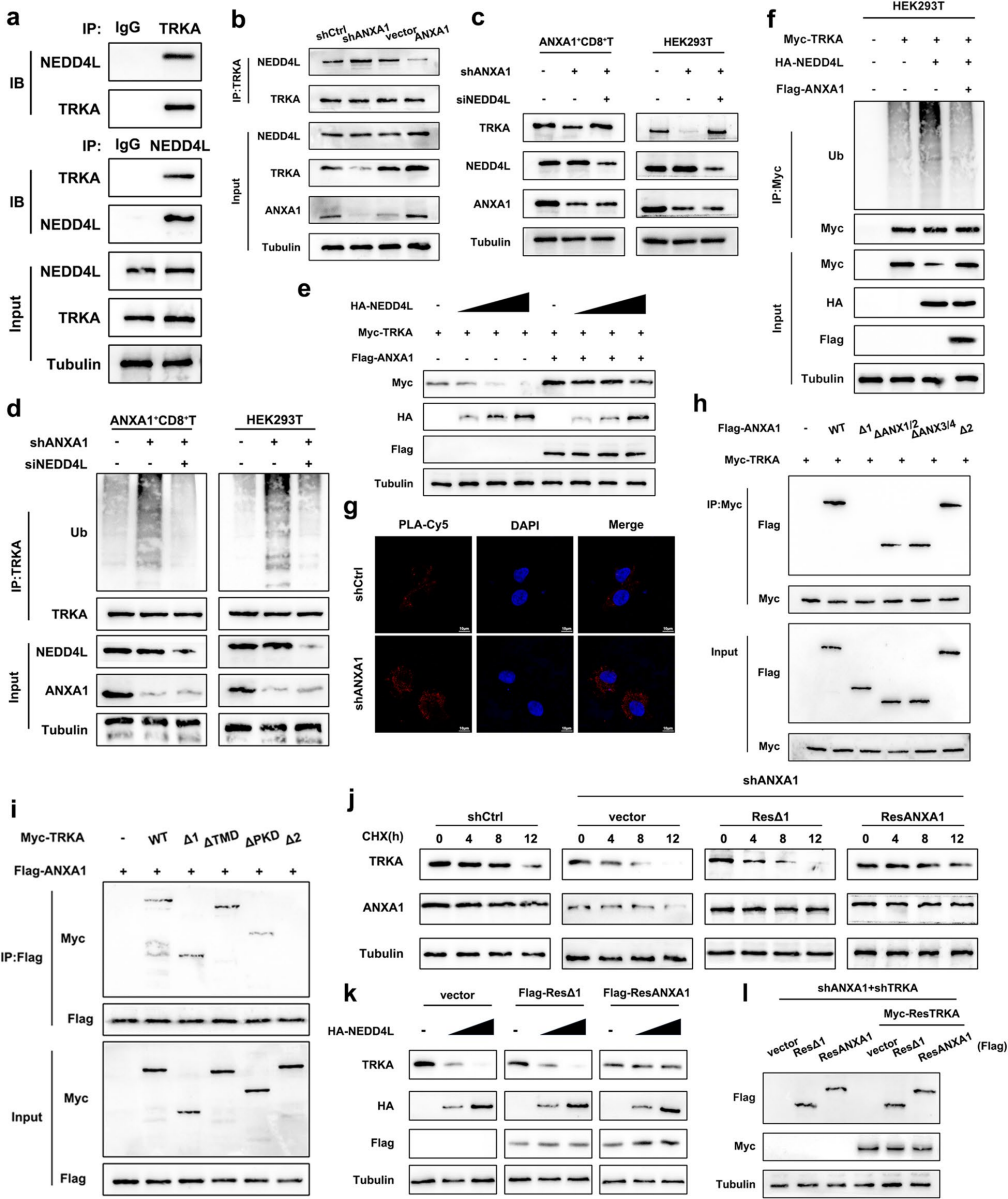
Competitive combination of ANXA1 and NEDD4L with TRKA. **a** Co-IP was conducted to verify the binding of TRKA to NEDD4L in ANXA1^+^CD8^+^T cells; **b** Co-IP was conducted to verify the effect of ANXA1 on the binding of TRKA and NEDD4L; **c** The effect of interference with NEDD4L on the downregulation TRKA expression caused by knocking down ANXA1; **d** Co-IP was conducted to verify of the effect of interference with NEDD4L on the increase ubiquitination level of TRKA caused by knocking down ANXA1; **e** The effects of different doses of NEDD4L and ANXA1 on TRKA protein levels; **f** Co-IP was conducted to verify the antagonistic effect of NEDD4L and ANXA1 on the ubiquitination level of TRKA; **g** Proximity Ligation Assay (PLA) was conducted to verify the effect of knocking down ANXA1 on the binding of NEDD4L and TRKA, scale bars:10 μm; **h** Co-IP was conducted with truncated plasmids to identify the binding region of ANXA1; **i** Co-IP was conducted with truncated plasmids to identify the binding region of TRKA; **j** The effect of ANXA1-Δ 1 (59-346aa of ANXA1) on the stability of TRKA protein; **k** The effect of ANXA1-Δ 1 (59-346aa of ANXA1) on the degradation of TRKA protein; **l** Re-transfection of different combinations of ANXA1, Δ 1, and TRKA after knockdown of ANXA1 and TRKA

Mass spectrometry following the immunoprecipitation of TRKA revealed the presence of the classical E3 ubiquitin ligase NEDD4L (Fig. S6a), which has been previously demonstrated to modulate the stability of tyrosine kinase receptors and facilitate the proteasomal degradation of TRKA [36]. Co-IP confirmed the interaction between TRKA and NEDD4L in ANXA1^+^CD8^+^T cells (Fig. 6a). Following the knockdown of ANXA1 in ANXA1^+^CD8^+^T cells, we observed a significant reduction in TRKA protein expression, accompanied by an evident increase in its association with NEDD4L. Conversely, the overexpression of ANXA1 had the opposite effect (Fig. 6b). Additionally, we noted that interfering with NEDD4L expression efficiently reversed the decreased TRKA expression and elevated ubiquitination levels induced by ANXA1 knockdown (Fig. 6c-d). Also, NEDD4L dose-dependently reduced TRKA protein levels and concurrently enhanced its ubiquitination, whereas ANXA1 antagonized the effects of NEDD4L on both the protein expression and ubiquitination state of TRKA (Fig. 6e-f). When ANXA1 was knocked down, an increased association between TRKA and NEDD4L was observed (Fig. 6g). Collectively, these findings indicated that ANXA1 enhanced TRKA protein stability by competitively binding to TRKA with NEDD4L.

To elucidate the specific regions involved in the interaction between ANXA1 and TRKA, a series of truncated plasmids for ANXA1 and TRKA were constructed (Fig. S6b-c). Immunoprecipitation demonstrated that the Δ1 truncation of ANXA1 failed to interact with TRKA and the Δ2 truncation of TRKA did not bind to ANXA1, suggesting that the N-terminal region of ANXA1 and the C-terminal region of TRKA are crucial for their mutual interaction (Fig. 6h-i). Subsequently, we investigated the impact of the N-terminal region of ANXA1 on TRKA stability. Re-expression of Δ1 did not restore TRKA protein stability after ANXA1 was knocked down, whereas reintroduction of full-length ANXA1 effectively reversed the decrease in TRKA levels (Fig. 6j). Similarly, full-length ANXA1, but not the Δ1 region of ANXA1, was able to counteract NEDD4L-induced TRKA degradation (Fig. 6k). These findings highlighted the pivotal role of the N-terminal domain of ANXA1 in maintaining TRKA stability.

We then examined the effect of the N-terminal region of ANXA1 on the function of ANXA1^+^CD8^+^ T cells. As shown in Fig. 6l, after ANXA1 and TRKA were knocked down in ANXA1^+^CD8^+^ T cells, we transfected various combinations of ANXA1, ANXA1-Δ1 (which cannot bind and stabilize TRKA), and TRKA plasmids. The results indicated that when Δ1 and TRKA were co-transfected, the expression levels of PD1 and TIGIT remained significantly reduced compared with those in the control group, while co-transfection of ANXA1 and TRKA restored PD1 and TIGIT expression levels, suggesting that the immunosuppressive state of ANXA1^+^CD8^+^ T cells was dependent on the interaction between ANXA1 and TRKA. (Fig. S6d-g). Notably, in the absence of TRKA expression, transfection with either ANXA1 or ANXA1-Δ1 could also increase the expression of exhaustion markers, suggesting that in addition to interacting with TRKA, ANXA1 may engage alternative targets to maintain the immunosuppressive phenotype of ANXA1^+^CD8^+^ T cells.

### Blocking the ANXA1/TRKA interaction reversed the exhausted function of ANXA1^+^CD8^+^T cells

These experiments substantiated that ANXA1 competed with NEDD4L for binding to TRKA, reducing TRKA ubiquitination and degradation and subsequently inducing exhaustion in ANXA1^+^CD8^+^ T cells. Hence, we speculated that interrupting the ANXA1–TRKA interaction could effectively inhibit ANXA1^+^CD8^+^T-cell exhaustion, suggesting a viable therapeutic strategy. Drawing from previous research [37], we engineered a small molecular peptide, A11, derived from the N-terminus of ANXA1 (amino acids 20–30): EYVQTVKSSKG. After the cells were incubated with 10 μM A11 for 24 h, immunofluorescence was used to confirm the intracellular penetration and functional efficacy of A11 (Fig. S7a). As expected, A11 effectively reduced the interaction between ANXA1 and TRKA while increasing the association of NEDD4L with TRKA (Fig. S7b). Moreover, A11 treatment led to an increase in the ubiquitination and degradation of TRKA (Fig. S7c-e), suggesting that A11 reduced the protein stability of TRKA. Immunofluorescence revealed an increase in the colocalization of TRKA with NEDD4L upon A11 treatment (Fig. S7f). Collectively, these findings confirmed that A11 impeded the competitive binding of ANXA1 to NEDD4L, consequently facilitating TRKA degradation.

We subsequently examined the effect of A11 on the function of ANXA1^+^CD8^+^T cells. After ANXA1^+^CD8^+^T cells were treated with A11, the glycolytic activity and protein expression of key glycolytic genes significantly increased (Fig. S7g-h), while the expression of exhaustion markers markedly decreased (Fig. S7i-j). These observations led us to concluded that the small-molecule peptide A11, derived from ANXA1, inhibited the functional exhaustion of ANXA1^+^CD8^+^ T cells by blocking the interaction between ANXA1 and TRKA.

### NGF-TRKA interaction induced an immunosuppressive status in ANXA1^+^CD8^+^T cell

**Fig. 7:**
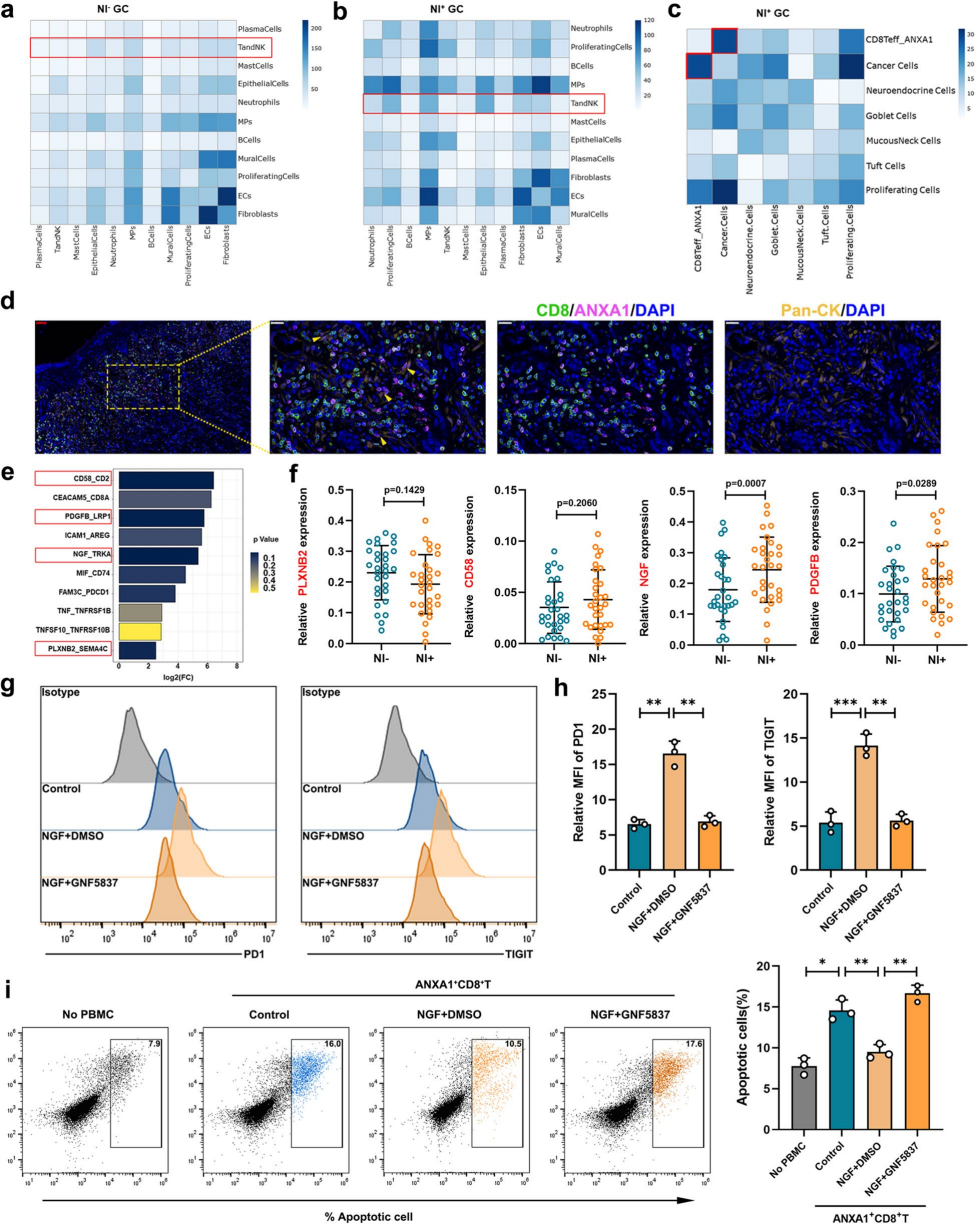
NGF-TRKA interaction induced an immunosuppressive status in ANXA1^+^CD8^+^T cell. **a-b** Heatmap representing the number of predicted ligand-receptor pairs between different cell types in NI^−^GC samples and NI^+^GC samples; **c** Heatmap representing the number of predicted ligand-receptor pairs between T-NK cells and epithelial cells in NI^+^GC samples; **d** Representative images of mIHC for ANXA1^+^CD8^+^T cells (ANXA1: purple; CD8: green) and cancer cells (pan-CK: yellow), the spatial association was denoted by yellow arrowheads; red scale bar:50 μm, white scale bar:20 μm; **e** Selected interactions between ANXA1^+^CD8^+^T cells and cancer cells (the top 10 mean values of the receptor/ligand pairs between two clusters); **f** The mRNA levels of CD58, PDGFB, NGF, PLXNB in gastric cancer tissues (*n* = 30); **g** Flow cytometry of the exhaustion factors in ANXA1^+^CD8^+^T cells after NGF/GNF5837 treatment; **h** Quantitative expression of the exhaustion factors in ANXA1^+^CD8^+^T cells after NGF/GNF5837 treatment; **i** The apoptosis rate of cancer cells co-cultured with ANXA1^+^CD8^+^T cells after NGF/GNF5837 treatment. GC cells and ANXA1^+^CD8^+^T cell were harvested from the same NI^+^GC patient. All experiments were repeated 3 times with consistent results. The data are presented as the means ± SD. *p*-values were determined by two-tailed unpaired Student’s t-test(f) and paired Student’s t-test(g-i). *p < 0.05, **p < 0.01, and ***p < 0.001 versus the control group

Having elucidated the alterations in infiltration, biological functions, and underlying molecular mechanisms of ANXA1^+^CD8^+^ T cells within the NI^+^GC microenvironment, we proceeded to further investigate their interactive relationships. Guided by an analysis from CellPhoneDB, we identified differential intercellular interactions and their respective expression levels between various cell types in NI^+^GC and NI^−^GC, which underscores the intricate complexity inherent in the tumor microenvironment. In the NI^−^GC samples, T and NK cells primarily interacted with epithelial cells, mononuclear phagocytes, endothelial cells, fibroblasts, and mural cells (Fig. 7a), whereas in the NI^+^GC samples, T and NK cells displayed closer interactions with proliferative cells, epithelial cells, and mononuclear phagocytes (Fig. 7b). These findings highlighted that the biological functions of T and NK cells are regulated by multiple cellular pathways within the tumor immune microenvironment. Interaction analysis further revealed an increased number of receptor‒ligand pairs between ANXA1^+^CD8^+^ T cells and tumor cells in the NI^+^GC cohort (Fig. 7c), which was corroborated by multiplex immunohistochemistry experiments (Fig. 7d).

In NI^+^GC, the CD58_CD2, PDGFB_LRP1, NGF_TRKA, and PLXNB2_SEMA4C interactions between ANXA1^+^CD8^+^T cells and tumor cells were particularly enhanced (Fig. 7e). We expanded our investigation to quantify the expression levels of CD58, PDGFB, NGF, and PLXNB in GC tissues. The expression of NGF (nerve growth factor) was significantly upregulated in the NI^+^GC group. (Fig. 7f). NGF, which is vital for neural development and function, also regulates tumor proliferation, resistance, and metabolism through the TRKA receptor. Recent reports have highlighted the role of NGF/TRKA signaling in modulating T-cell immunoresponsiveness in melanoma, with inhibition of this axis demonstrating synergistic effects in combination with immunotherapy [38]. Nonetheless, its impact on GC immunity remains poorly understood. NGF was added to ANXA1^+^CD8^+^ T cells to validate the effects of NGF. Our findings revealed that NGF significantly increased the expression of exhaustion markers in ANXA1^+^CD8^+^ T cells (Fig. 7g-h) and reduced apoptosis of cocultured cancer cells (Fig. 7i). However, pretreatment with the TRKA inhibitor GNF5837 effectively counteracted the effects of NGF. These results suggested that NI^+^GC induced an immunosuppressive state in ANXA1^+^CD8^+^ T cells via the NGF–TRKA interaction, reinforcing the pivotal role of TRKA in maintaining the immunosuppressive status of ANXA1^+^CD8^+^ T cells.

### ANXA1/TRKA axis facilitated immune evasion in gastric cancer

**Fig. 8:**
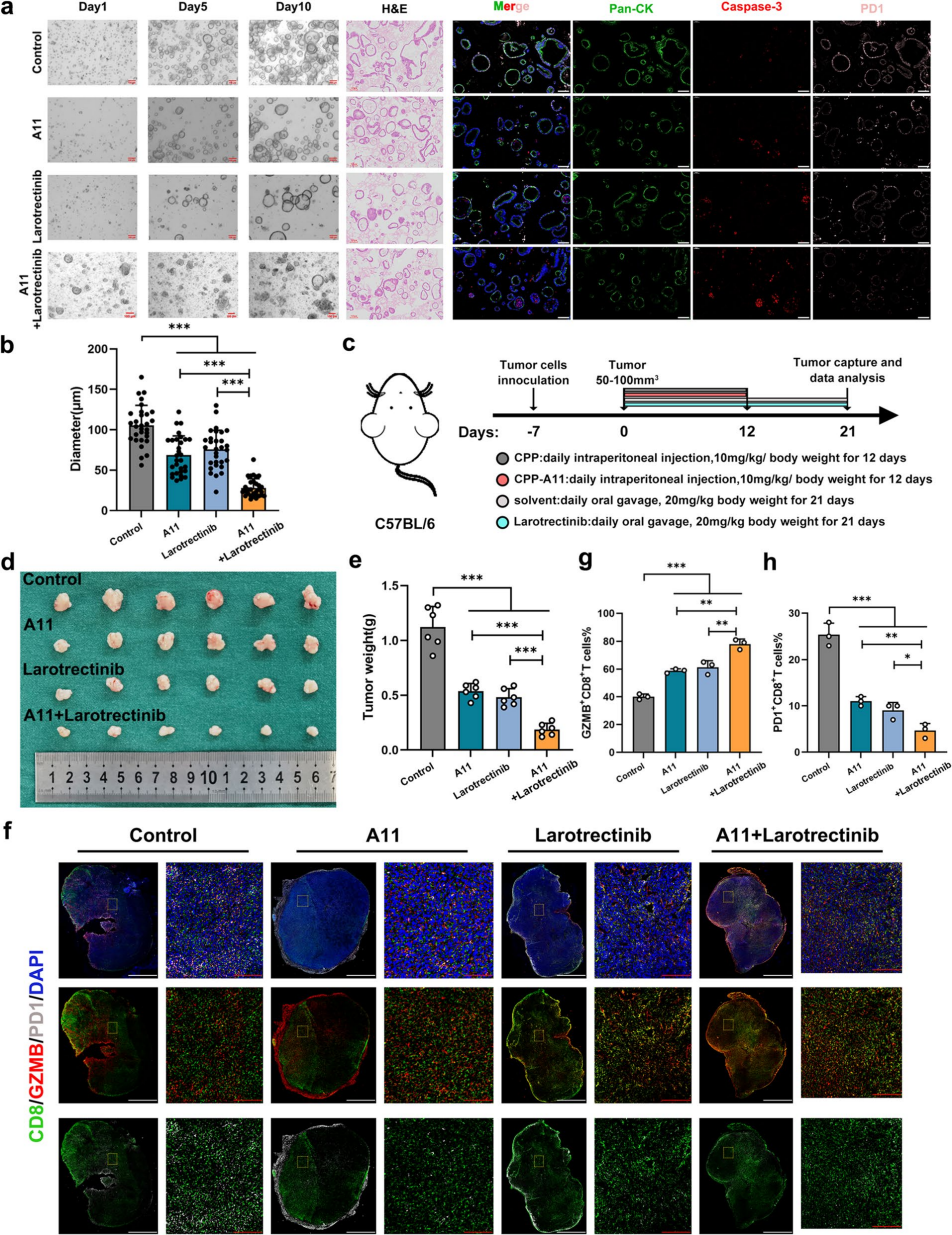
ANXA1/TRKA axis facilitated immune evasion in gastric cancer. **a** Representative images of growth (left, scale bar:100 μm), H&E (middle, scale bar:100 μm), and mIHC (right, pan-CK: green; caspase-3: red; PD1: pink, scale bar:100 μm) of patient derived organoids (PDOs) derived from NI^+^GC patient, with the addition of A11 and/or TRKA antibody-Larotrectinib; **b** Statistics of organoids after adding A11/Larotrectinib; **c** Schematic diagram exhibiting the grouping and treatment plan of the C57BL/6 mouse model; **d-e** Tumor weight of subcutaneous xenografts in indicated groups is shown, *n* = 6; **f** MIHC (CD8: green; GZMB: red; PD1: grey) of subcutaneous xenografts in indicated groups, white scale bar:50 mm, red scale bar:100 μm; **g-h** The percentage of GZMB^+^CD8^+^T cells and PD1^+^CD8^+^T cells in indicated groups; The data are presented as the means ± SD. *p*-values were determined by two-tailed unpaired Student’s t-test. *p < 0.05, **p < 0.01, and ***p < 0.001 versus the control group

To further elucidate the impact of ANXA1/TRKA on GC immunity, patient derived organoids (PDOs) were established from NI^+^GC samples and cocultured with cognate ANXA1^+^CD8^+^T cells. Treatment with A11 or Larotrectinib notably inhibited the growth of PDOs, with a more profound effect observed in response to both treatments. Immunofluorescence were performed on organoids collected from the respective groups, revealing a significant increase in apoptotic levels within the GC organoids following treatment with A11 or Larotrectinib (Fig. 8a-b). In addition, xenograft tumor models in mice were established, followed by treatment with A11 and Larotrectinib (Fig. 8c). Tumors were harvested four weeks later, and the results demonstrated that A11 and Larotrectinib also inhibited tumor growth in vivo. (Fig. 8d-e). Subsequent multiplex immunohistochemical staining confirmed that A11 and Larotrectinib increased effector T-cell infiltration while effectively reducing the infiltration of exhausted T cells, and the effect was more significant when A11 and Larotrectinib were combined (Fig. 8f-h).

Additionally, we further explored the effect of targeting the ANXA1/TRKA axis on immunotherapy sensitivity. Coculture experiments in vitro revealed that the secretion of effector factors by ANXA1^+^CD8^+^ T cells significantly increased when the anti-PD1 antibody was combined with A11 and Larotrectinib (Fig. S8a-d), and the killing effect on tumor cells was also significantly enhanced compared with that of monotherapy (Fig. S8e-f). To further confirm the in vitro findings, we established an in vivo NI model in which GC cells were implanted at the periphery of the sciatic nerve. After 4 weeks, the sciatic nerve was surrounded by xenograft tissue. After combined treatment, tumor growth was significantly inhibited (Fig. S8g-h), accompanied by increased proportions of GZMB^+^CD8^+^T cells and Perforin^+^CD8^+^T cells (Fig. S8i-k). These results suggested that combination therapy effectively enhanced the response to immunotherapy and inhibited tumor growth. The above results also suggest that targeting the ANXA1/TRKA axis may effectively improve the response rate to immunotherapy in NI^+^GC patients.

In summary, these findings validated the role of ANXA1/TRKA in promoting immune evasion in GC, suggesting promising therapeutic potential for the combined use of A11 and anti-TRKA antibody in GC immunotherapy as a novel therapeutic approach.

## Discussion

As a significant pathological characteristic, NI is frequently observed in various cancers, with incidence rates ranging from 6.8% to 75.6% among oncologic patients. In the case of GC, a meta-analysis of 24 studies involving 30,590 GC patients who underwent curative gastrectomy revealed a median rate of NI positivity of 40.9% (ranging from 6.8% to 75.6%) [39]. Our previous research identified NI in 270 out of 592 GC patients, representing 45.61% of the cohort [11]. NI is associated with aggressive tumor behavior, recurrence, neoplastic pain, and poor survival outcomes across multiple tumor types. In our study, we found that NI correlated with various critical features of advanced GC, including tumor size, location, depth of invasion, lymph node metastasis, TNM stage, D dissection, tumor differentiation, Lauren classification, and blood vessel invasion. Furthermore, we also found that NI was associated with overall survival. Understanding the distinct immunological characteristics of NI^+^GC is crucial for developing targeted treatment strategies and enhancing therapeutic outcomes for this specific subtype.

ScRNA-seq overcomes the limitations of bulk RNA-seq by resolving cell-type-specific transcriptional profiles, enabling precise characterization of cellular heterogeneity in complex diseases like GC. This approach reveals distinct functional states and identifies diagnostic/prognostic markers within GC tissues, directly informing early diagnosis and treatment response prediction. In this study, scRNA-seq analysis of NI^+^GC and NI^−^GC through cluster annotation, differential expression, and cell–cell interaction profiling identified significantly elevated proportions of mononuclear phagocytes, mast cells, neutrophils, T/NK cells, plasma cells, B cells, and proliferating cells in NI⁺GC samples.

Tumor cells express abnormal antigens presented via MHC-I, enabling CD8⁺T cells to recognize and eliminate them through cytotoxic granules (perforin/granzyme B) and cytokines (IFN-γ、TNF-α). However, chronic antigen exposure drives CD8⁺T cell exhaustion—characterized by impaired proliferation, cytotoxicity, and cytokine secretion alongside sustained inhibitory receptor expression [40]. Within the tumor microenvironment, immunosuppressive tactics (coinhibitory molecules, immunosuppressive cytokines, hypoxic/nutrient-deprived conditions) further disable tumor-infiltrating CD8⁺T cells. Consequently, CD8⁺T cell subsets exhibit functional heterogeneity in cytotoxic capacity, cytokine profiles, metabolism, and inhibitory receptor expression [29]. Given the direct correlation between CD8⁺T cell functionality and tumor progression and patient prognosis, deciphering this heterogeneity is essential for advancing personalized immunotherapy, efficacy prediction, and prognostic assessment in cancer.

Single-cell UCell scoring is a method used to assess the activity of specific gene sets within individual cells. By algorithmically scoring gene sets related to signaling pathways, functional modules, or disease markers, the expression and potential functional state of these gene sets in every single cell can be determined [41]. After determining that the cytotoxic UCell scores of T and NK cells were lower in the NI^+^GC group than in the NI^−^GC group, the results of multiplex immunofluorescence in tissue microarray and in vitro functional assays also suggested that CD8^+^ T cells in the NI^+^GC group were in a state of functional exhaustion. Further clustering analysis and subpopulation annotation were performed on single T and NK cells, and we identified and validated the specifically upregulated ANXA1^+^CD8^+^ T-cell population in NI^+^GC, which was in the exhausted state. Pathway enrichment analysis of differentially expressed genes and metabolic pathway scoring indicated that the underlying mechanism of ANXA1^+^CD8^+^ T-cell dysfunction may be related to aberrant glucose metabolism.

T cells, as pivotal components of the immune system, require substantial energy upon activation to support their proliferation, differentiation, and execution of effector functions. Glucose metabolism plays a central role in this process. In their resting state, T cells primarily rely on oxidative phosphorylation (OXPHOS) for energy production; however, upon activation, to meet rapidly increasing energy demands, T cells swiftly switch their metabolic mode to aerobic glycolysis to generate ATP, even in the presence of ample oxygen—a phenomenon known as the Warburg effect. This process involves increased glucose uptake, increased activity of key glycolytic enzymes, and elevated lactate production. Abnormal glucose metabolism in CD8^+^ T cells can directly affect their survival, proliferation, and effector functions. It has been confirmed that targeting key enzymes involved in glucose metabolism can augment the antitumor immunity of CD8^+^ T cells [42, 43]. Our experiments revealed that ANXA1^+^CD8^+^ T cells exhibited lower glucose metabolic activity than ANXA1^−^CD8^+^ T cells did, which explained the diminished effector function and exhausted state of ANXA1^+^CD8^+^ T cells.

Protein‒protein interactions (PPIs), which are fundamental to numerous biological processes, regulate protein stability, subcellular localization, and cell cycle progression through specific binding between two or more proteins and can serve as key targets for treating diseases. Abnormal PPIs can lead to disrupted signaling pathways, abnormal enzyme activity, and improper protein folding and degradation, significantly contributing to cancer development [44, 45]. Small-molecule peptides, as bioactive molecules, are structurally diverse and can mimic the critical binding regions of natural proteins, competitively binding to target proteins and disrupting their normal interactions. Designed small-molecule peptides can intervene in PPIs by blocking ligand binding, stabilizing inactive conformations, or promoting dissociation, making them effective anticancer agents through disruption of these interactions. In this study, the small-molecule peptide A11, derived from the N-terminus of ANXA1, disrupted the ANXA1–TRKA interaction while promoting the binding of NEDD4L to TRKA, thereby reducing TRKA expression and inhibiting immune escape. Our findings revealed a critical role for a novel small-molecule peptide in curbing tumor immune escape.

CellPhoneDB analysis, which is based on a database of known ligand–receptor pairs, infers potential intercellular communication networks by analyzing the expression levels of ligand and receptor genes across different cell types. It is a bioinformatic approach used to explore intercellular interactions in scRNA-seq data [46]. We found that ANXA1^+^CD8^+^ T cells had more receptor‒ligand interactions with tumor cells in NI^+^GC samples. Further screening and validation revealed that the NGF–TRKA interaction was the most prominent. NGF, a member of the neurotrophic factor family, binds to the high-affinity transmembrane receptor TRKA, which not only plays a crucial role in the development and maintenance of neuronal functions such as growth, differentiation, protection, regeneration, and pain transmission but also participates in malignant behaviors, including proliferation, invasion, metastasis, and drug resistance, leading to poor prognosis in cancer patients. The addition of exogenous NGF indicated that NI^+^GC induced an immunosuppressive state in ANXA1^+^CD8^+^ T cells through NGF_TRKA, increasing our understanding of the microenvironment in NI^+^GC. These findings further underscore the central role of TRKA in ANXA1^+^CD8^+^ T cells.

Immunotherapy has become a critical component of GC treatment, offering new options for advanced disease. Checkpoint inhibitors such as nivolumab and pembrolizumab are now approved for PD-L1-positive or microsatellite instability-high (MSI-H) GC, acting by blocking PD-1/PD-L1 interactions to restore T-cell antitumor activity. Despite this progress, significant limitations persist: biomarkers like PD-L1 expression, tumor mutational burden, and MSI lack predictive precision for patient selection, no unified screening standard exists, and acquired resistance frequently develops through compensatory immunosuppressive pathways or microenvironment remodeling. Additionally, immune-related adverse events (irAEs) pose substantial clinical challenges. Although immunotherapy improves objective response rates and disease control compared to chemotherapy, most patients remain non-responsive, underscoring the urgent need for robust biomarkers and rational combination strategies to enhance therapeutic efficacy. Larotrectinib, a tyrosine kinase inhibitor (TKI) targeting neurotrophic receptor tyrosine kinases (NTRKs), is primarily indicated for patients harboring NTRK gene fusions without known resistance mutations. It effectively inhibits the growth and spread of tumor cells through NTRK gene fusion, promotes tumor cell apoptosis, and ultimately achieves effective tumor treatment [47]. Proven in preclinical trials to synergize with existing immunotherapies, it has been approved by FDA as a targeted therapy for patients with TRK translocations [48]. Given the importance of ANXA1 in stabilizing TRKA and the antitumor immune activity of A11, further evaluation of the safety and efficacy of combining A11 with anti-TRKA antibodies is warranted to determine whether it is expected to synergize with GC immunotherapy.

The study also has some remaining limitations, including a relatively small cohort size. Further validation in larger patient cohorts and functional studies is needed to confirm these novel insights. Beyond T cells, other critical cellular components within the NI^+^GC tumor microenvironment, such as myeloid-derived suppressor cells and tumor-associated macrophages, indispensably orchestrate the immunosuppressive landscape and drive tumor progression, warranting further mechanistic investigation.

To summarize, our study provides the first comprehensive characterization of the immune microenvironment in NI^+^GC at the single-cell level, revealing that increased infiltration of ANXA1⁺CD8⁺T cells is correlated with a poor prognosis in patients with gastric cancer. While prior studies have established ANXA1's role in fostering an immunosuppressive tumor microenvironment, through mechanisms such as regulatory T cell recruitment and natural killer cell suppression, our work reveals a novel metabolic axis by which ANXA1 drives immune evasion. Specifically, ANXA1 stabilizes TRKA protein levels to suppress glycolytic activity and effector functions in CD8⁺T cells. This mechanism establishes ANXA1⁺CD8⁺T cells as critical architects of the immunosuppressive niche in NI^+^GC. Critically, our findings position targeting the ANXA1/TRKA axis as a promising immunotherapeutic strategy for NI^+^GC.

## Materials and methods

### Clinical samples

In this study, clinical samples comprising tissue and blood specimens were obtained from patients at the Gastric Cancer Center of the First Affiliated Hospital of Nanjing Medical University, who had not received preoperative chemotherapy or radiotherapy and were confirmed to have no other metastatic lesions. The collection and use of these samples were approved by the Ethics Committee of the First Affiliated Hospital of Nanjing Medical University(2024-SR-557). All patients or their guardians provided written informed consent after being fully informed. Detailed records of each patient's specific information, including basic data and postoperative pathological information, such as age, gender, date of surgery, tumor location, TNM stage, neural invasion, and vascular invasion, were meticulously documented.

### Mouse xenograft models

C57BL/6 mice aged between four to five weeks were obtained from GemPharmatech (Nanjing, China) and housed within a Specific Pathogen-Free (SPF) grade animal facility (temperature: 20–22℃, relative humidity: 61–65%, 12 h light/dark cycle, five mice per cage). The mice had free access to tap water and standard commercial mouse chow. Subcutaneous injection of MFC cells at a concentration of 10^6^ cells in 100 μl is administered to the mice. Post-inoculation, the cohort were randomized into four distinct groups: the control group; the CPP-A11 treated group, receiving intraperitoneal injections of CPP-A11 at 10 mg/kg daily for twelve consecutive days; the TRKA antibody treated group, orally administered Larotrectinib at 20 mg/kg daily for a period of three weeks; and a combination treatment group of CPP-A11 and TRKA antibody. Daily monitoring and recording of tumor dimensions (volume calculated as length × width squared divided by two) ensue. Upon the conclusion of the treatment regimens, mice were euthanized, and the xenograft tumors were excised. Measurements and data pertaining to the tumors were meticulously recorded, and the excised tumors were preserved in fixative solutions, earmarked for subsequent immunohistochemistry and immunofluorescence studies.

### Reagents and antibodies

ImmunoCult Human CD3/CD28 T Cell Activator (10,971) was purchased from STEMCELL, Cycloheximide (HY-12320), Larotrectinib (HY-12866) were purchased from MedChemExpress, MG132 (S2619) was purchased from Selleck, Apoptosis Detection Kit (C1065M) was purchased from Beyotime. Antibodies are shown in Supplementary Table 2.

### Cell culture

Human cell lines AGS and HEK293T used in this study were purchased from the cell bank of the Chinese Academy of Science, and tested by short tandem repeat (STR) cell identification to eliminate cell pollution. Cells were maintained in high-glucose DMEM, supplemented with 10% fetal bovine serum (FBS) and 100 U/mL penicillin–streptomycin. All cells were grown at 37℃ in a humidified incubator with 5% CO2. All cell lines used in this study were regularly tested by mycoplasma PCR detection and confirmed to be free of mycoplasma contamination.

### Statistical analysis

The data were analyzed using GraphPad Prism 9.0 software (RRID:SCR_002798) and represented as mean ± SD for replicates. The sample size, statistical methods, and relevant details are provided in the Figure legends, main text, or methods section. The statistical tests were performed as two-sided. Results of cell culture experiments were collected from at least 3 independent biological replicates. The differences within two groups were compared by unpaired Student’s t test, paired Student’s t test or nonparametric Wilcoxon rank-sum test as indicated. The Pearson correlation test was used to examine the significant correlation between quantitative variables. Kaplan–Meier curves (log rank test) were used for survival analysis. *P < 0.05, **P < 0.01, and ***P < 0.001 versus the corresponding controls were indicated.

## Supplementary Information

Supplementary Material 1.

## Data Availability

All data needed to evaluate the conclusions are included in this article and/or in its supplemental material. The raw sequence data reported in this paper have been deposited in the Genome Sequence Archive (Genomics, Proteomics & Bioinformatics 2025) in National Genomics Data Center (Nucleic Acids Res 2025), China National Center for Bioinformation/Beijing Institute of Genomics, Chinese Academy of Sciences (GSA-Human: HRA016041) that are publicly accessible at https://ngdc.cncb.ac.cn/gsa-human.
