# Supplementary material for: Targeting ANXA1/TRKA axis enhances immunotherapy sensitivity in neural invasion-positive gastric cancer
Source: Mol Biomed. 2026 Apr 9;7:48. doi: 10.1186/s43556-026-00444-1 (PMC13065939; doi:10.1186/s43556-026-00444-1)
Supplement: Supplementary file 1 — Supplementary Material 1. [file 43556_2026_444_MOESM1_ESM.docx]

**Targeting ANXA1/TRKA axis enhances immunotherapy sensitivity in neural invasion-positive gastric cancer**

Tianlu Jiang^#1,2,3^, Peng Zhou^#4^, Yikai Shen^#2,3^, Jie Lin^#2,3^, Ying Li^5^, Xusheng Shen^2,3^, Lang Fang^2,3^, Penghui Xu^2,3^, Zekuan Xu^*2,3,6,7^, Linjun Wang^*2,3^, Yiwen Xia^*2,3^

^1^Department of General Surgery, The Affiliated Wuxi People’s Hospital of Nanjing Medical University, Wuxi People’s Hospital, Wuxi Medical Center, Nanjing Medical University, Wuxi, Jiangsu Province, China.

^2^Gastric Cancer Center, The First Affiliated Hospital of Nanjing Medical University, Nanjing, Jiangsu Province, China

^3^Department of General Surgery, The First Affiliated Hospital of Nanjing Medical University, Nanjing, Jiangsu Province, China

^4^ Department of General Surgery, Jiangnan University Medical Center, Wuxi, Jiangsu Province, China

^5^Hepatobiliary Surgery, Department of General Surgery, Huashan Hospital & Cancer Metastasis Institute, Fudan University, Shanghai, China.

^6^Institute for Gastric Cancer Research, Nanjing Medical University, Nanjing, Jiangsu Province, China

^7^Jiangsu Key Lab of Cancer Biomarkers, Prevention and Treatment, Collaborative Innovation Center for Personalized Cancer Medicine, Nanjing Medical University, Nanjing, Jiangsu Province, China

**^*^Corresponding authors:**

**Yiwen Xia**: xiayiwennjmu@163.com

**Linjun Wang**: wanglinjun@njmu.edu.com

**Zekuan Xu**: xuzekuan@njmu.edu.cn

**^#^These authors contributed equally to this work: Tianlu Jiang, Peng Zhou, Yikai Shen, Jie Lin**

**Supplementary materials**

**Supplementary material 1: Supplementary Figures**

**Supplementary Figure1:**

**UMAP plot color-coded (gray to orange) to represent the expression levels of the marker genes for the eleven cell types, which are listed beyond the UMAP plot.**


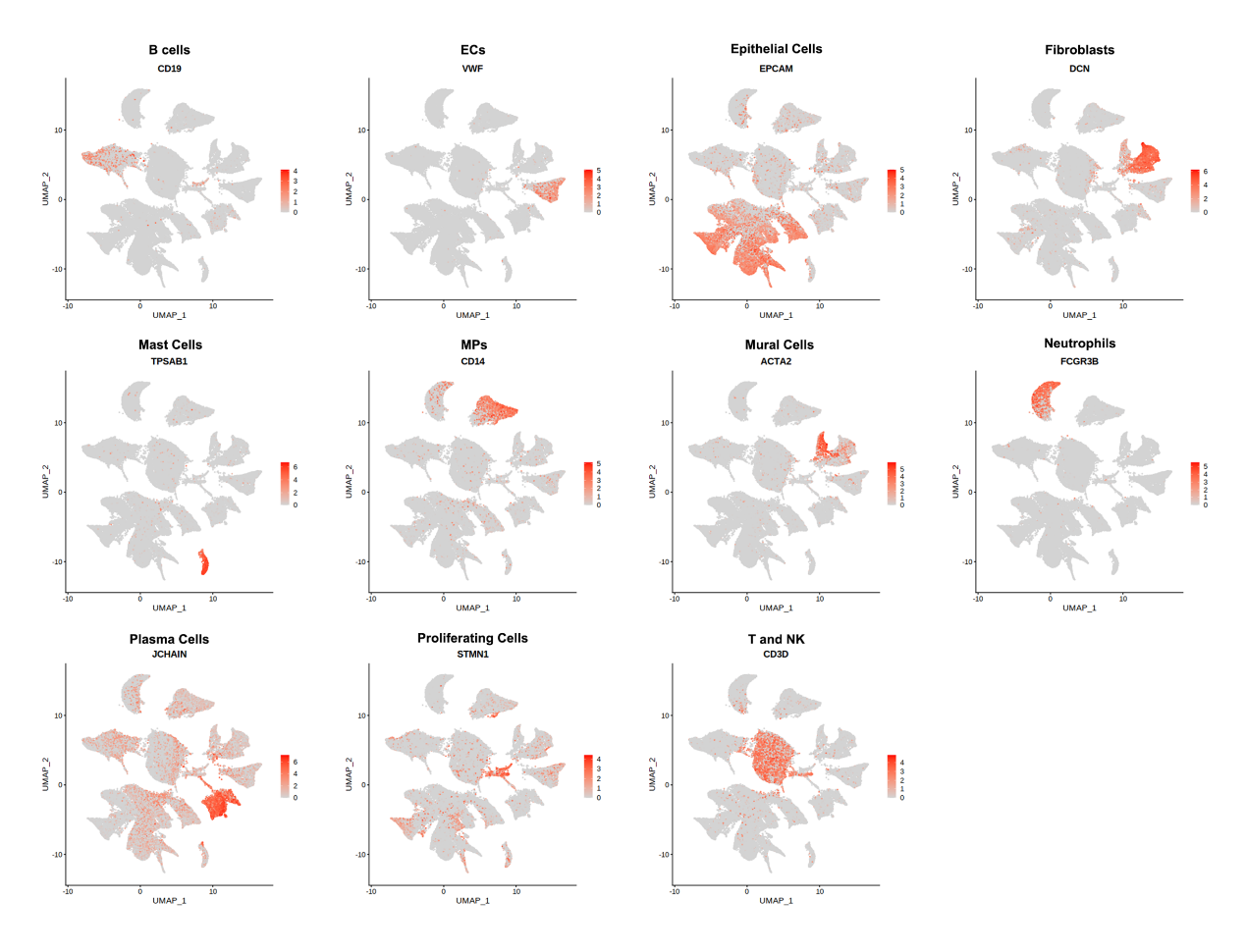


**Supplementary Figure 2 The functional status of CD8^+^T cells in NI^+^GC and NI^-^GC**


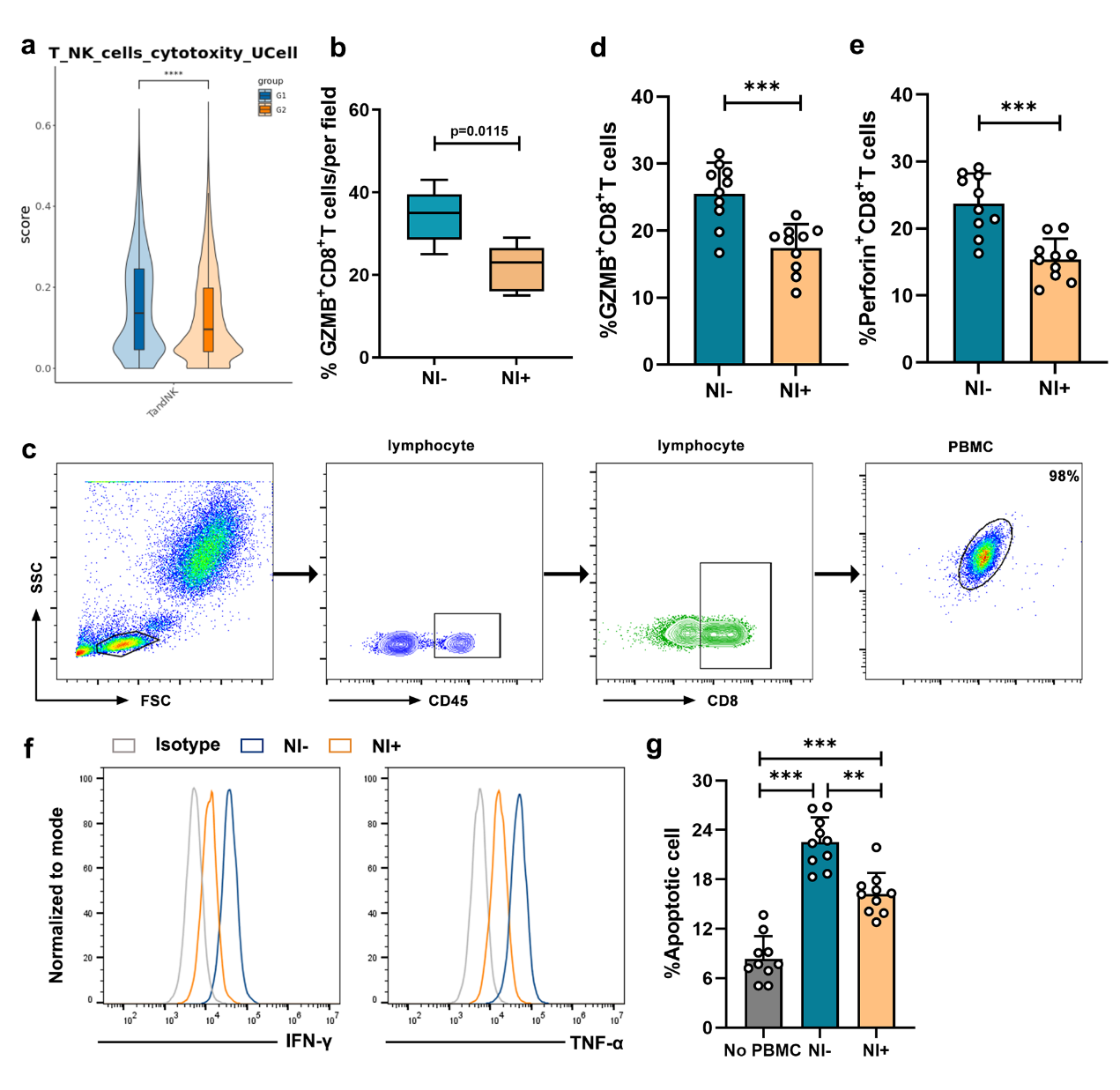


**a** UCell scoring of T and NK cell subpopulation. (G1: NI^-^GC, G2:NI^+^GC); **b** Statistics of the percentage of GZMB^+^CD8^+^T cells in NI^-^GC and NI^+^GC microarrays; The centerlines indicate the median. The box limits indicate the first and third quartiles. The whiskers indicate the maxima and minima; **c** Sorting process of CD8^+^T cells in peripheral blood; **d-e** The percentage of GZMB^+^CD8^+^T cells and Perforin^+^CD8^+^T cells in NI^-^GC and NI^+^GC (n=10); **f** Flow cytometry of IFN-γ and TNF-α by intracellular staining in NI^-^GC and NI^+^GC; **g** The apoptosis rate statistics of GC cells co-cultured with CD8^+^T cells derived from NI^-^GC and NI^+^GC patients (n=10).The data are presented as the means ± SD. *p*-values were determined by two-tailed unpaired Student’s *t*-test. **p <* 0.05, ***p <* 0.01, and ****p <* 0.001 versus the control group.

**Supplementary Figure 3 The exhausted state of the upregulated ANXA1^+^CD8^+^T cells in NI^+^GC**


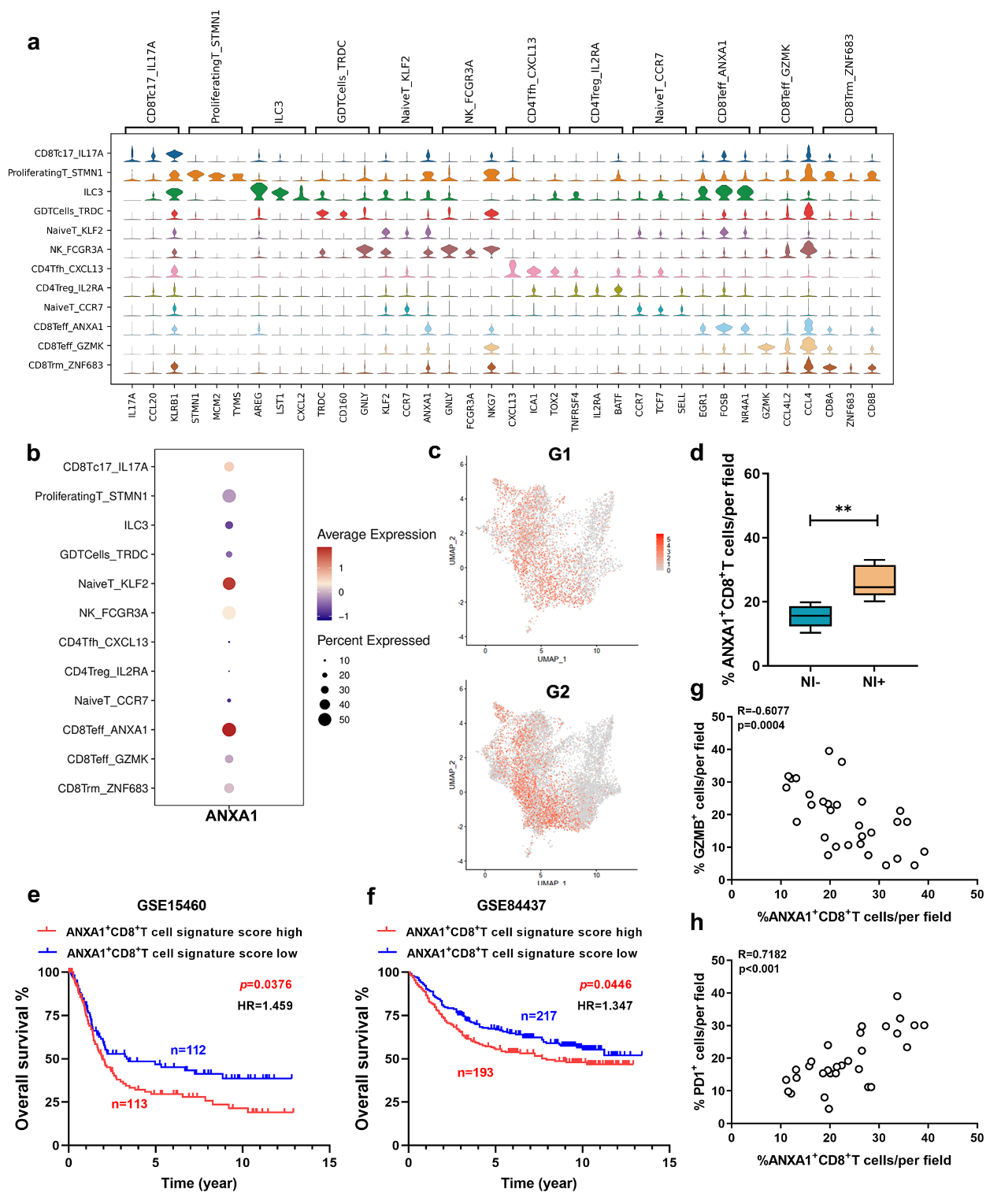


**a** Violin diagram of canonical marker genes expressed in each T and NK cell cluster; **b** Dot plot of ANXA1 in all identified T and NK cell clusters in single cell dataset. Color represents average expression. Size of the dot represents expression frequency; **c** Feature plot of ANXA1 expression in all identified T and NK cell clusters in gastric cancer (G1: NI^-^GC, G2:NI^+^GC). Low expression is in grey, while high expression is in orange; **d** Statistics of the percentage of ANXA1^+^CD8^+^T cells in NI^-^GC and NI^+^GC (n=30); The centerlines indicate the median. The box limits indicate the first and third quartiles. The whiskers indicate the maxima and minima; *p*-values were determined by two-tailed unpaired Student’s *t*-test. ***p <* 0.01 versus the control group; **e-f** Survival analysis of GC patients with different infiltration of ANXA1^+^CD8^+^T cells in GSE15460 and GSE84437. The signature of ANXA1^+^CD8^+^T cells based on the expression of characteristic gene cluster, including NR4A1, FOSB, EGR1, FOS, HSPH1, CITED2, AREG, NFKBIA, JUN, and ANXA1; **g-h** Correlation analysis of the percentage of ANXA1^+^CD8^+^T cells and GZMB^+^ cells, PD1^+^ cells in NI^+^GC (n=30).

**Supplementary Figure 4 ANXA1^+^CD8^+^T cells exhibited diminished glycolytic activity**


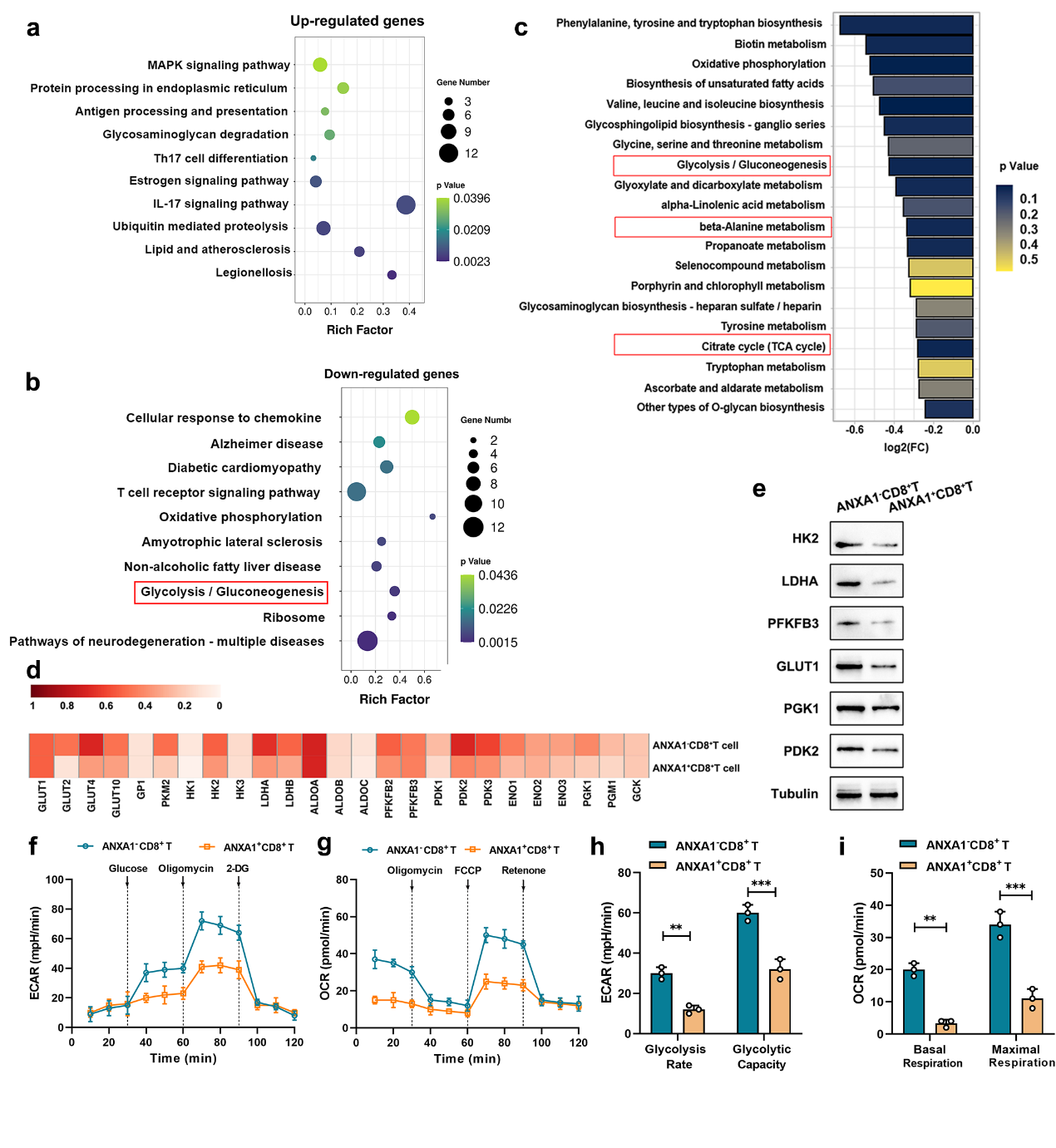


**a-b** Kyoto Encyclopedia of Genes and Genomes (KEGG) pathway analyses of differentially expressed genes (DEGs) highly and lowly expressed in ANXA1^+^CD8^+^T cells; the size indicates the gene numbers, and the color indicates the p values; **c** Metabolic related pathways enriched in ANXA1^+^CD8^+^T cells estimated by GSVA; **d-e** The mRNA and protein levels of key glycolytic genes in ANXA1^+^CD8^+^T cells and ANXA1^-^CD8^+^T cells; **f-g** ECAR assays and OCR assays to detect the glucose metabolism activity of ANXA1^+^CD8^+^T cells and ANXA1^-^CD8^+^T cells; **h** Glycolysis rate and glycolytic capacity in ANXA1^+^CD8^+^T cells and ANXA1^-^CD8^+^T cells; **i** Basal respiration and maximal respiration in ANXA1^+^CD8^+^T cells and ANXA1^-^CD8^+^T cells. All experiments were repeated 3 times with consistent results. The data are presented as the means ± SD. *p*-values were determined by two-tailed unpaired Student’s *t*-test. **p <* 0.05, ***p <* 0.01, and ****p <* 0.001 versus the control group.

**Supplementary Figure 5 ANXA1 inhibited glucose metabolism and induced the exhausted function of ANXA1^+^CD8^+^T cells depending on TRKA.**


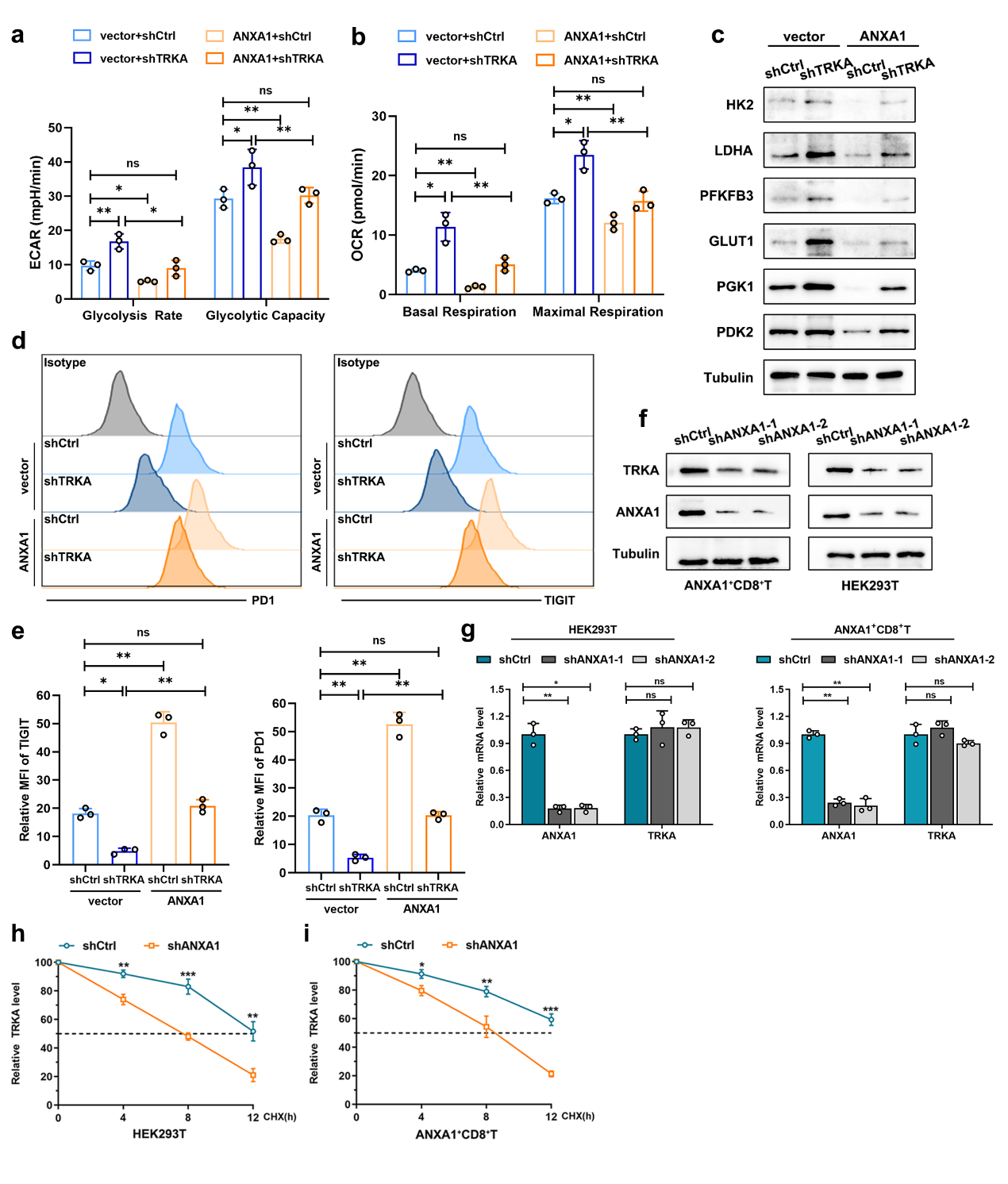


**a-b** ECAR assays and OCR assays to detect the glucose metabolism activity of indicated groups; **c** Protein expression of key genes involved in glycolysis in indicated groups; **d-e** Flow cytometry and quantification of the exhaustion factors (PD1 and TIGIT) in indicated groups; **f-g** The protein and mRNA level of TRKA after knocking down ANXA1; **h-i** Quantitative expression of TRKA protein after CHX treatment in HEK293T cells and ANXA1^+^CD8^+^T cells. All experiments were repeated 3 times with consistent results. The data are presented as the means ± SD. *p*-values were determined by two-tailed paired Student’s *t*-test. **p <* 0.05, ***p <* 0.01, and ****p <* 0.001 versus the control group.

**Supplementary Figure 6 The immunosuppressive state of ANXA1^+^CD8^+^T cell was dependent on the interaction between ANXA1 and TRKA**


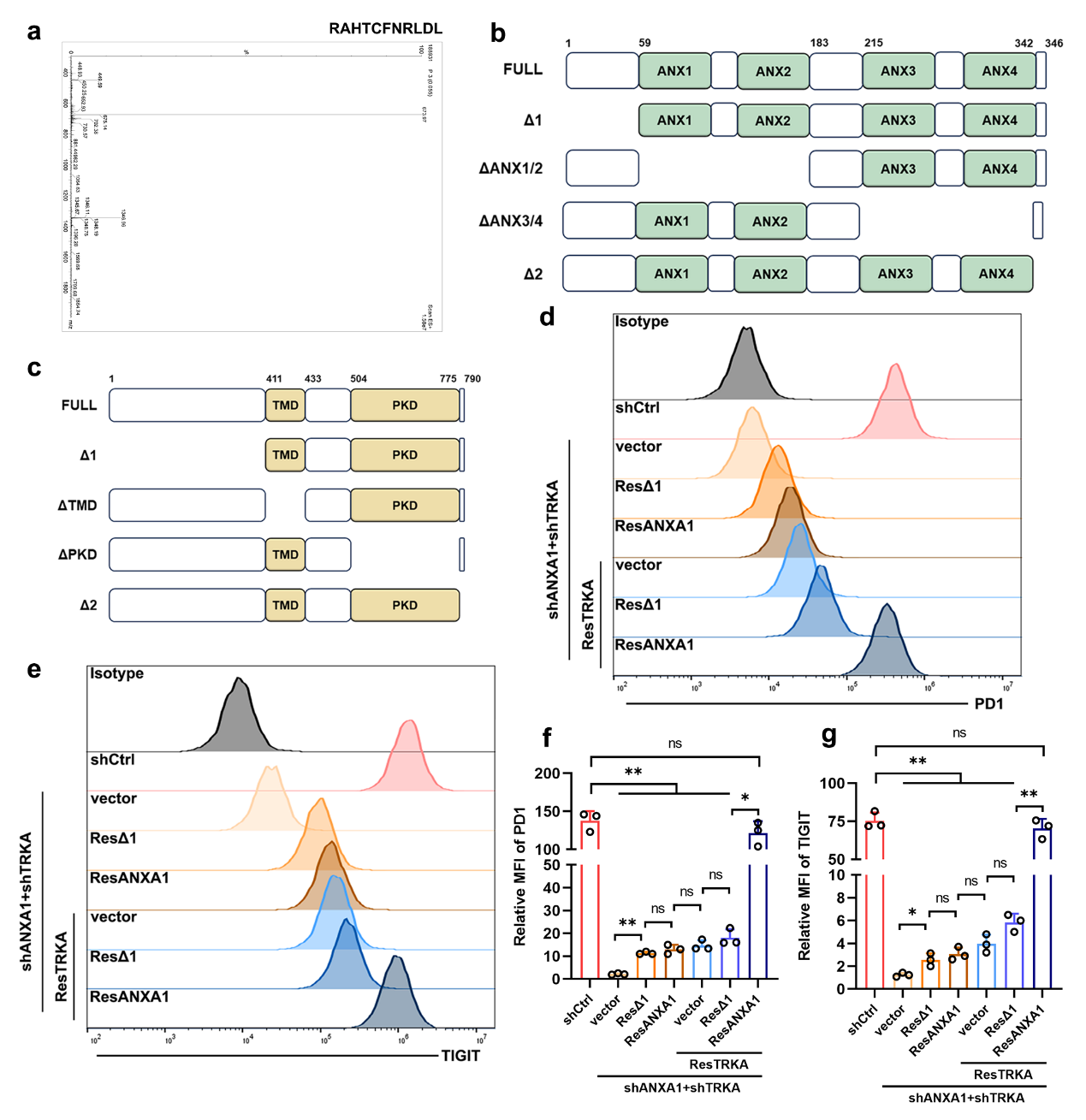


**a** Mass spectrum of NEDD4L; **b** Schematic diagram of ANXA1 and its truncate mutants, ANX: [Annexin](https://www.uniprot.org/keywords/KW-0041) domain; **c** Schematic diagram of TRKA and its truncate mutants, TMD: transmembrane domain; PKD: [protein kinase](https://www.uniprot.org/uniprotkb?query=%28family%3A%22protein+kinase+superfamily%22%29) domain; **d-g** Expression levels of PD1 and TIGIT in ANXA1^+^CD8^+^T cells of different treatment groups. All experiments were repeated 3 times with consistent results. The data are presented as the means ± SD. *p*-values were determined by two-tailed paired Student’s *t*-test. **p <* 0.05, ***p <* 0.01, and ****p <* 0.001 versus the control group.

**Supplementary Figure 7 Blocking ANXA1/TRKA interaction reversed the exhausted function of ANXA1^+^CD8^+^T cells**


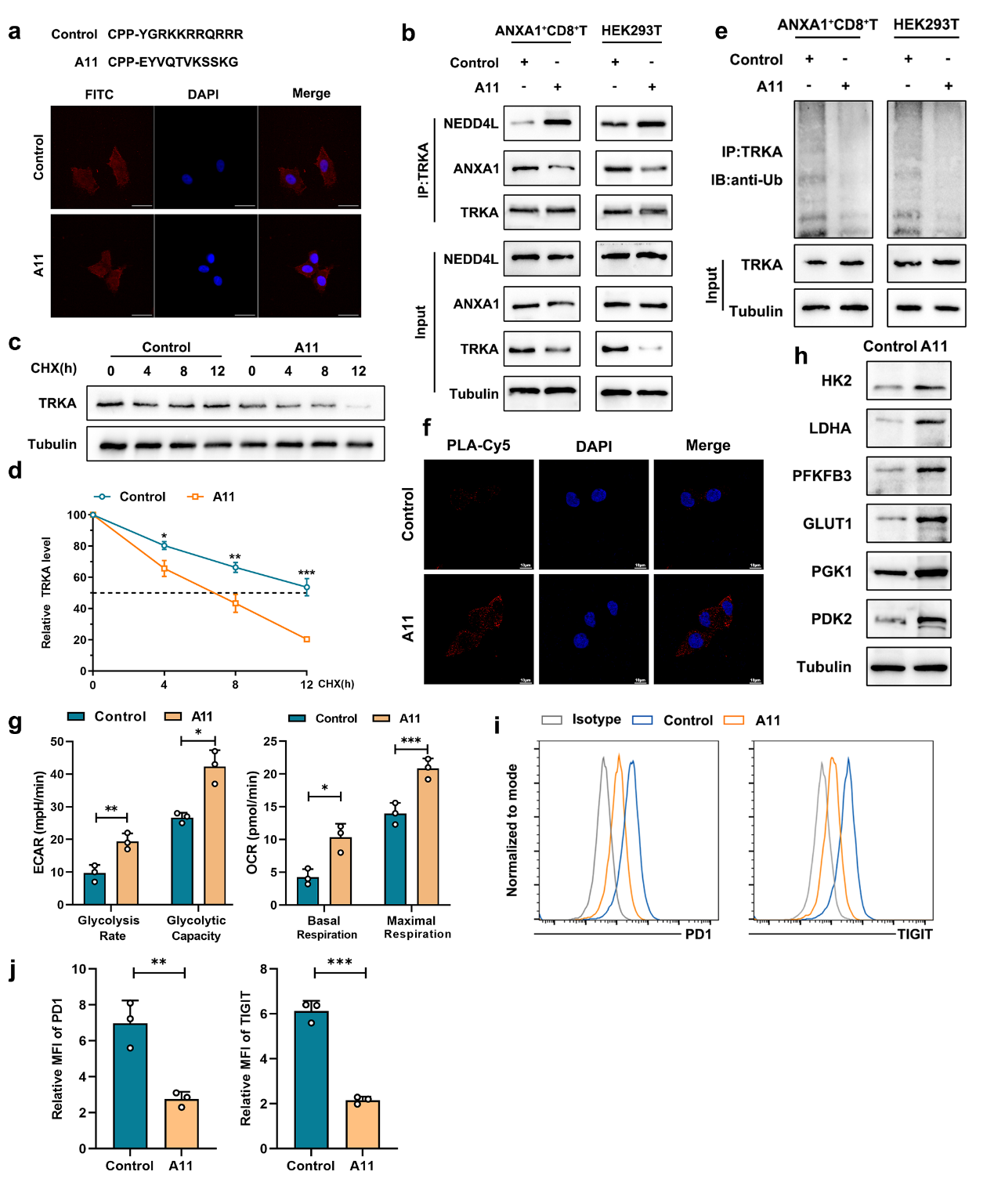


**a** Confocal microscopy of ANXA1^+^CD8^+^T cells staining for the small molecular peptide A11 derived from the N-terminus of ANXA1 (red). Scale bar:20μm; **b** The effect of A11 on the competitive binding of TRKA between ANXA1 and NEDD4L; **c-e** The effect of A11 on the stability and ubiquitination level of TRKA protein; **f** PLA assay was conducted to verify the effect of knocking down A11 on the binding of NEDD4L and TRKA, scale bar:10μm; **g** Quantification of glycolysis and oxidative respiration in ANXA1^+^CD8^+^T cells treated with A11 was determined by seahorse assay. ECAR: extracellular acidification rate, OCR: oxygen consumption rate; **h** Protein expression of key genes involved in glycolysis in ANXA1^+^CD8^+^T cells treated with A11; **i-j** Flow cytometry and quantitative expression of the exhaustion factors (PD1 and TIGIT) in ANXA1^+^CD8^+^T cells after A11 treatment. All experiments were repeated 3 times with consistent results. The data are presented as the means ± SD. *p*-values were determined by two-tailed paired Student’s *t*-test. **p <* 0.05, ***p <* 0.01, and ****p <* 0.001 versus the control group.

**Supplementary Figure 8 Targeting ANXA1/TRKA axis enhances immunotherapy sensitivity in NI^+^GC**


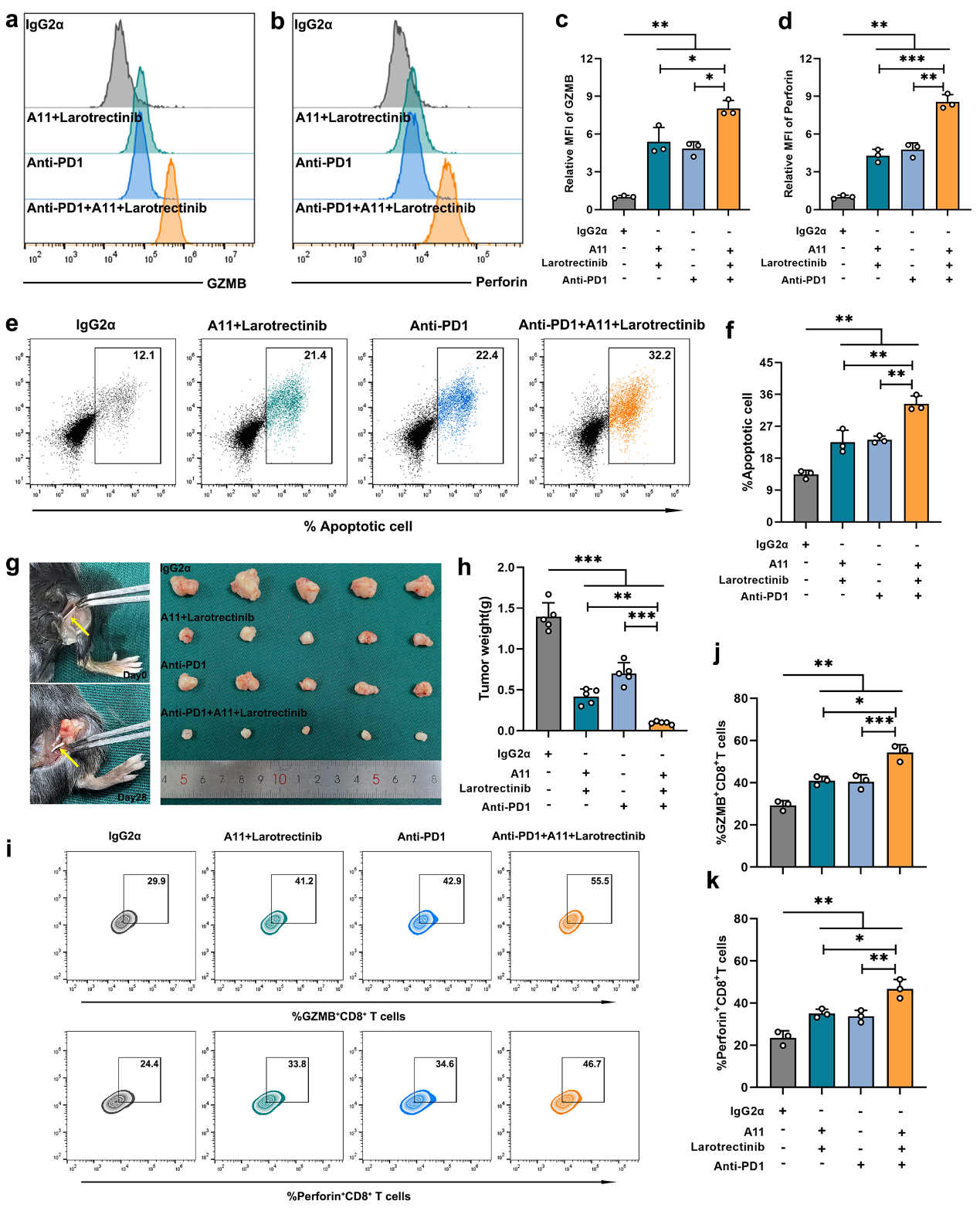


**a-d** Flow cytometry and quantitative expression of the effector factors (GZMB、Perforin) in ANXA1^+^CD8^+^T cells after A11+ Larotrectinib and/or anti-PD1 antibody treatment; **e-f** The apoptosis rate of cancer cells co-cultured with ANXA1^+^CD8^+^T cells after A11+ Larotrectinib and/or anti-PD1 antibody treatment. GC cells and ANXA1^+^CD8^+^T cell were harvested from the same NI^+^GC patient. **g** Left: Anatomical images showed the experimental design of sciatic nerve tumor invasion model. 4 weeks after implanting GC cells along the perineurium of the sciatic nerves, the sciatic nerves were surrounded by xenografts. Yellow arrow points to the sciatic nerve. Right: The images of xenograft tumors from sacrificed mice of *in vivo* NI model.n=5 **h** Tumor weight of xenograft tumors of sacrificed mice of *in vivo* NI model. All experiments were repeated 3 times with consistent results. **i-k** Flow cytometry and quantification of GZMB^+^CD8^+^T cells and Perforin^+^CD8^+^T cells from the harvest tumors in indicated groups; The data are presented as the means ± SD. *p*-values were determined by two-tailed paired Student’s *t*-test. **p <* 0.05, ***p <* 0.01, and ****p <* 0.001 versus the control group.

**Supplementary material 2: Supplementary methods**

**Extraction single-cell suspension from gastric cancer tissue**

Gastric cancer tissue samples were collected under sterile conditions and washed with pre-chilled PBS to remove blood contamination, then rapidly transported to the laboratory at 4°C. After washed separately in 75% ethanol and antibiotics, gastric tissues were minced into a chyle-like consistency and digested in a water bath shaker at 37°C; after filtration and centrifugation, the single-cell suspension of tumor tissue was obtained. The suspension was slowly overlaid onto the upper layer of gradient lymphocyte separation medium along the side of the tube, then the lymphocytes were collected from the intermediate layer and resuspended in lymphocyte culture medium for further cultivation.

**Isolation and activation of peripheral blood mononuclear cells**

According to the manufacturer's instructions, peripheral blood mononuclear cells (PBMCs) were isolated from peripheral blood using lymphocyte separation medium. The diluted blood was carefully layered on top of the lymphocyte separation medium. Following centrifugation, the mononuclear cell layer was aspirated, washed, and counted. Activation was induced by adding IL-2 and ImmunoCult XF T Cell Expansion Medium supplemented with ImmunoCult Human CD3/CD28 T Cell Activator. For further expansion, fresh complete ImmunoCult XF T Cell Expansion Medium containing IL-2 was added to the cell suspension, adjusting the viable cell density to 1*10^6^/mL every 2-3 days.

**Immunohistochemistry (IHC)**

Firstly, the paraffin-embedded slides were dewaxed and hydrated, followed by antigen repair using a high-temperature water bath. After blocking with 10% goat serum, the tissue sections were incubated overnight at 4°C with the PGP9.5 antibody. Post multiple washes with buffer, secondary antibodies corresponding to the species origin of the primary antibody were applied for incubation. A brown precipitate was generated by the chromogen 3,3'-diaminobenzidine (DAB) to visualize the target protein (PGP9.5), with counterstaining of cell nuclei using hematoxylin to enhance the localization and expression pattern discernment. For the evaluation of immunohistochemical staining, a total score is calculated by combining the assessment of staining intensity (negative: 0, weak: 1, moderate: 2, strong: 3) with the percentage of immunopositive tumor cells (0%: 0, 1%-25%: 1, 26%-50%: 2, >50%: 3), yielding values ranging from 0 to 6. Two pathologists independently score each sample to minimize bias and ensure reproducibility of the results.

**Multiplex immunohistochemical staining (mIHC)**

Initially, the paraffin sections were baked at 60°C for 30 minutes, followed by sequential immersions in xylene and absolute ethanol twice for 5 minutes each, then through a gradient of alcohols (from 95% to 70%) for 5 minutes each, and finally rinsed with water and PBS. Subsequently, antigen retrieval was performed by microwaving the sections in citrate or EDTA buffer, ensuring that the tissue remained submerged throughout the process. After cooling to room temperature, the sections were washed three times in PBS. The tissue sections were blocked in blocking solution at room temperature for 30 minutes, then covered with diluted primary antibodies and incubated overnight at 4°C, with the addition of a small amount of water in the humidified box to prevent antibody evaporation. Post incubation, the sections were washed thrice in PBS, followed by incubation with diluted HRP-conjugated secondary antibodies for 1 hour at room temperature, and another series of PBS washes. Fluorescent dyes were then diluted 1:200 in buffer and applied to the samples for an incubation period of 10-15 minutes. This process was repeated for the staining of additional antibodies, with fluorescent visualization at each round's end. Nuclear counterstaining was achieved by covering the sections with DAPI and incubating them in the dark for 10 minutes, followed by three PBS washes. After removing excess liquid, the sections were mounted using a fluorescence quenching mounting medium. Observation and recording were carried out using the Thunder Imager high-resolution inverted fluorescence microscope. Throughout this meticulous process, the goal was to achieve multiplex immunolabeling and precise detection with high resolution imaging.

**Single-cell RNA sequencing (scRNA-seq)**

Approximately 0.5 grams of fresh gastric cancer tissue samples were collected, washed with pre-chilled PBS to remove surface impurities, then fully submerged in tissue preservation solution. Properly labeled and sealed, they were stored and transported under conditions of 2~8°C. Sample dissociation and subsequent machine operation tasks were entrusted to Nanjing Newgene Bioinformatics Technology. The data preprocessing phase encompassed quality control, UMI processing, assessment of mitochondrial gene ratios in cells, cell filtration, and data normalization. Utilizing techniques for dimensional reduction and visualization allowed for the intuitive presentation of high-dimensional gene expression data. Hierarchical clustering algorithms were employed to delineate cell subpopulations. Cell type annotation was based on the identification of marker genes, determining cell types through database comparisons. Subsequent analyses included differential gene expression, functional enrichment (GO/KEGG), pseudotime analysis to explore the dynamics of cell differentiation, and intercellular communication network analysis (ligand-receptor interaction) among other customized analyses, providing a comprehensive interpretation of cellular community characteristics and their biological significance.

**Isolation, activation, and cultivation CD8^+^T cells**

To isolate and activate CD8^+^T cells via immunomagnetic bead separation, start by washing the beads conjugated with anti-CD8 and resuspending them in buffer for standby. Next, mix the prepared single-cell suspension with the beads in a specific ratio, using a magnetic stand to separate CD8^+^T cells, followed by washing and resuspension steps, ultimately collecting purified CD8^+^T cells. Activation involves co-culturing these cells with CD3/CD28 immunomagnetic beads under appropriate conditions, whereupon the activated CD8^+^T cells can be used for subsequent experiments, with the removal of beads when necessary. For the expansion of CD8^+^T cells, culture them in a medium containing growth factor, serum, and antibiotics, regularly inspecting cell condition and density, passing them at the right time to maintain optimal growth conditions. When cell viability declines, re-stimulate the CD8^+^T cells with CD3/CD28 immunomagnetic beads, ensuring the accuracy of experimental results and cell health. Throughout the process, continuous monitoring and timely adjustments were critical to ensure effective cell culture outcomes.

**Apoptosis assay**

CD8^+^T cells were cocultured with gastric cancer cells at an optimal ratio in a 96-well plate, nourished with complete medium supplemented with serum, dual antibiotics, rIL-2, and anti-CD3/28 T cell activators. Post a designated co-culture duration, the cells and medium were discarded, followed by cleansing with pre-chilled PBS. Subsequently, trypsin was introduced to digest gastric cancer cells until adequately processed, post which cells were harvested and subjected to two rounds of washing with pre-chilled PBS. Upon completion of washing, cells were resuspended in buffer, followed by the introduction of Annexin V and PI, and incubated in the dark for 15 minutes. Throughout this phase, undyed negative controls and single-stain controls were established. Conclusively, cell apoptosis levels were quantified via flow cytometry. This methodological approach was meticulously designed to appraise the nuanced impact of CD8^+^T cells on the apoptotic fate of gastric cancer cells.

Flow cytometry

CD8^+^T cells were seeded in a 96-well plate and cultivated in complete medium enriched with serum, dual antibiotics, rIL-2, and anti-CD3/28 T cell activators. Thereafter, CD8^+^T cells cocultured with gastric cancer cells were harvested, centrifuged, and washed with PBS, before being resuspended in complete medium. Re-stimulation was initiated by the addition of PMA, Ionomycin, and Golgistop, followed by a 4-6hour incubation period. Post centrifugation and PBS washing, cells underwent fixation and permeabilization, then were incubated with fluorescently labeled antibodies targeting specific effector molecules in the dark for 30 minutes. Negative, isotype, and single-stain controls were concurrently set up. Post-incubation, cells were washed to eliminate unbound antibodies and resuspended in buffer at an appropriate ratio. Ultimately, cytokine expression levels were quantified on a flow cytometer, a meticulous process aimed at assessing the functional activity of CD8^+^ T cells.

**Western blot**

First, the lower gel solution was blended with buffer, to which accelerator was added uniformly, poured into the mold, and overlaid with isopropanol for surface leveling. Upon solidification of the lower gel—evident by a distinct demarcation line with the isopropanol—the alcohol was discarded. Subsequently, the upper gel solution was prepared, comb teeth inserted, and left to solidify prior to commencing electrophoresis. Electrophoresis buffer, transfer buffer, and TBST buffer were formulated. Protein samples along with markers were loaded into the gel lanes, undergoing constant voltage electrophoresis at 160v until proteins neared the bottom of the lower gel. Prior to transfer, PVDF membranes were activated in methanol, then sequentially layered with the components, immersed in transfer buffer, and subjected to a constant current of 400mA for blotting. Following transfer, PVDF membranes were blocked in blocking solution for an hour, washed with TBST, and incubated with primary antibodies overnight at 4°C, followed by three TBST washes. Secondary antibodies were then incubated for 2 hours at room temperature, again followed by three TBST washes. Final visualization was achieved using a chemiluminescence imager, with images preserved for subsequent analysis, encapsulating a meticulous workflow aimed at proficient protein expression analysis.

**Quantitative Real-time PCR（qRT-PCR）**

Following the manufacturer's instructions, total RNA was extracted from tissue samples using TRIzol reagent. Concurrently, total RNA was efficiently isolated from cultured cell lines using TRI Reagent. Subsequently, the harvested RNA was converted into complementary DNA (cDNA) via reverse transcription, setting the stage for subsequent analysis. The execution of quantitative Real-Time Reverse Transcription PCR (qRT-PCR) relied on the Roche Light Cycler detection system and SYBR RT-PCR kit, enabling precise quantification of specific gene expression levels. Throughout this process, the GAPDH gene served as a standard reference, ensuring accurate data normalization. Employing the 2^(-ΔΔCT)^ method, we scientifically evaluated the fold change in target transcript abundance relative to the reference group. The specific primer sequences utilized in this qRT-PCR experiment were detailed below. The final experimental outcomes represent the mean values of at least three independent replicates.

**Plasmids construction and transfection**

The plasmids employed were custom-engineered by miaolingbio (Wuhan, China). Genes encoding ubiquitin and its various mutants—specifically K6, K11, K27, K29, K33, K48, K63, and the K48R variant—were synthetically produced by Sangon Biotech (Shanghai, China). These genes were meticulously inserted into either pcDNA3.1-HA or pcDNA3.1-V5 mammalian expression vectors. The accuracy of all genetic constructs was verified through DNA sequencing to ensure fidelity. For transfection purposes, Lipofectamine^TM^ 3000 reagent was deployed following the protocols outlined by the manufacturer. Cells were then permitted to proliferate for an additional 48 hours before undergoing selection with puromycin, a process that extended over one week. The efficacy of the transfection procedure was subsequently assessed utilizing qRT-PCR and Western blot analyses to confirm successful gene expression.

**Construction of stable cell lines**

For knocking down *ANXA1* expression, the target sequences (shRNA: 5'- GCAACCATCATTGACATTCTT-3') were cloned into the pLKO.1-TRC vector using *BamH I* and *EcoR I* sites. The negative control sequence was 5’-CCTAAGGTTAAGTCGCCCTCG-3’. ShANXA1 and control virus were designed and synthesized by OBiO Technology (Shanghai, China). For lentivirus packaging, HEK-293T cells were seeded in a 100 mm dish and transfected with pMD2.G, psPAX2 and constructed shRNA vectors mixed in a ratio of 1:3:4 to form a DNA transfection reagent complex. After 8 hours, the medium was removed and complete medium was added for another 48 hours. The lentivirus in the supernatant was filtered through 0.45μm membrane and storaged at -80 ℃. The virus titer was ≥ 10 ⁶ TU/mL to ensure subsequent infection efficiency. After treating 6-well plates with Retronectin (10 μ g/mL, incubated at room temperature for 1 hour), pre-packaged lentivirus and cells were centrifugated at a low-speed (300g, 30 minutes) and then added into 6-well plates for lentiviral infection. IL-2 (100 IU/mL) was necessary to prevent cell apoptosis.

**Proximity Ligation Assay**

Duolink^®^ PLA assay kit was used to detect the protein-protein interaction. The experiment began by on fixation and permeabilization of slides. Each squared centimeter received a drop of blocking solution, incubated at 37°C for an hour. Post-blocking, primary antibodies (anti-ANXA1, anti-TRKA, anti-NEDD4L), conjugated with oligonucleotides and optimized in concentration, were applied under conditions favorable to antibody binding. After incubation, slides underwent three 5-minute washes with 1x Wash Buffer A, followed by a ligation step using diluted ligation buffer and enzyme, incubated at 37°C for 30 minutes. This was succeeded by removal of the ligation mix, repeating the washing process, and an amplification step with polymerase, also incubated at 37°C but for 100 minutes. Following amplification, slides were washed again, detection solution added, and incubated at 37°C for 30 minutes. The protocol concludes with two 10-minute washes in 1x Wash Buffer B, a quick rinse in 0.01X buffer, and mounting using Duolink^®^ Mounting Media with DAPI, ready for fluorescence or confocal microscopy analysis after a 15-minute rest. Throughout, critical steps require light protection, and proper thawing and dilution of reagents were essential.

**Co-Immunoprecipitation (Co-IP)**

Magnetic beads were primed by gentle washing and resuspension in 1x TBS, setting them up for subsequent steps. Cells were then subjected to thorough lysis in a buffer supplemented with inhibitors, and the resultant lysate was clarified by centrifugation, retaining the supernatant for further manipulation. Diluted antibodies were introduced to Protein G magnetic beads, engaging in a 30-minute rotation to facilitate binding; post-binding, beads were washed and held in suspension. The antibody-bead complex was merged with the protein sample, embarking on an overnight incubation at 4°C to allow for immunoprecipitation. Following this extended interaction, beads underwent a series of washes in lysis buffer with inhibitors, ensuring removal of nonspecific bindings. Proteins were then liberated from the beads through the addition of 1x SDS buffer, followed by a 10-minute boiling step. A quick spin-down yields the supernatant, ready for immediate application in downstream assays or storage at -20°C for future use. Throughout, it was imperative to incorporate IgG and Input controlled to substantiate the validity of the experimental findings.

**Organoid models**

Under sterile conditions, gastric cancer tissue specimens were swiftly procured and rinsed with chilled PBS to cleanse residual blood from the surface. Subsequently, the tissues were immersed in transport medium and kept at 4°C during transit to the laboratory, accompanied by meticulous documentation of sample details. Upon arrival, the tissues were meticulously cleansed in primary culture buffer, minced to a fine texture, and exposed to primary tissue digestion solution. This ensued a 30-minute incubation in a 37°C water bath with intermittent agitation until individual cells or clusters became discernible under the microscope. Digestion was halted by filtration through a sieve, followed by centrifugation (4°C, 300g, 5min) to pellet the cells, a process repeated for thorough rinsing. Thereafter, cells were amalgamated with matrix gel, spread onto wells of a 24-well plate on ice, and placed in a 37°C incubator to solidify. Once solidified, organoid culture medium was added to initiate cultivation. Transitioning to passaging, the spent medium was discarded, replaced with chilled passage buffer, and left undisturbed for 2 minutes. The matrix gel was gently disrupted, and the suspension was collected, resting for an additional 10 minutes. Centrifugation (4°C, 300g, 5min) preceded treatment with organoid passage digestion solution for 2 minutes, followed by neutralization with passage buffer and another round of centrifugation. Cells were then resuspended in fresh matrix gel and plated according to the initial methodology for continued growth. Regarding cryopreservation, the culture medium was removed, and the organoids were briefly submerged in chilled passage buffer for 2 minutes prior to collection. Two rounds of centrifugation (4°C, 300g, 5min) and resuspension in passage buffer were executed, culminating in the addition of organoid cryopreservation medium. Cells were gently mixed, subjected to controlled rate cooling, and ultimately stored in liquid nitrogen for long-term preservation.

***In vivo* murine sciatic nerve model of NI**

A cell suspension of 10^6 MFC cells mixed with Matrigel (1:1, total volume 50μL) was injected around the sciatic nerve of the 6-week-old male C57BL/6 mice instead of injecting cell deposits directly into the sciatic nerve to simulate the microenvironment of tumor cell growth around the nerve as well as to facilitate better observation of the tumor size. 7 days after tumor inoculation, tumor-bearing mice were randomly assigned into four groups (n = 5 per group): vehicle control, anti-PD1 treatment (10 mg/kg, twice per week for 3 weeks), A11 and Larotrectinib treatment, the combined treatment. The mice were euthanized after 3 weeks. Tumor growth was monitored after harvesting, and the tumor size was determined by caliper measurements. The tumor volume was calculated using the formula: Tumor volume = length × width^2^ × 0.5.

**Supplementary material 3: Supplementary Tables**

**Supplementary Table1: The clinical characteristics of six gastric cancer patients analyzed in this study**

| Patients ID | Gender | Age | Tumor  location |  | Surgery  time | T | N | Pathological Stage | Vascular invasion | Neural invasion |
| --- | --- | --- | --- | --- | --- | --- | --- | --- | --- | --- |
| GC-1 | M | 73 | Corpus ventriculi |  | 2022/07 | 3 | 1 | IIB | P | N |
| GC-2 | M | 74 | Cardia |  | 2022/07 | 4a | 3a | IIIB | P | P |
| GC-3 | F | 52 | Corpus ventriculi |  | 2022/11 | 4a | 3b | IIIC | P | N |
| GC-4 | F | 48 | Corpus ventriculi |  | 2022/11 | 4a | 3b | IIIC | P | P |
| GC-5 | M | 72 | Cardia |  | 2022/12 | 4a | 3a | IIIB | P | N |
| GC-6 | M | 69 | Corpus ventriculi |  | 2022/12 | 3 | 1 | IIB | P | P |

M: Male; F: Female; P: positive; N: negative

**Supplementary Table2: Antibodies**

| **Antibodies** | **Source** | **Identifier** |
| --- | --- | --- |
| IgG2b isotype | BioXcell | BP0090 |
| IgG2a isotype | BioXcell | BE0089 |
| anti-PD-1 Antibody | BioXcell | BE0146 |
| anti-HA | Thermo Fisher Scientific | 26183 |
| anti-Flag | Cell Signaling Technology | 14793 |
| anti-GAPDH | Cell Signaling Technology | 5174 |
| [PerCP anti-human CD3 Antibody](https://www.biolegend.com/en-us/products/fitc-anti-human-cd3-antibody-3644) | BioLegend | 317306 |
| [FITC anti-mouse CD3 Antibody](https://www.biolegend.com/en-us/products/fitc-anti-mouse-cd3-antibody-45) | BioLegend | 100204 |
| [APC anti-human CD8 Antibody](https://www.biolegend.com/en-us/products/apc-anti-human-cd8-antibody-6531) | BioLegend | 344722 |
| [PerCP/Cyanine5.5 anti-mouse CD8 Antibody](https://www.biolegend.com/en-us/products/percp-cyanine5-5-anti-mouse-cd8b2-antibody-17484) | BioLegend | 140418 |
| [FITC anti-human CD45 Antibody](https://www.biolegend.com/en-us/products/fitc-anti-human-cd45-antibody-707) | BioLegend | 304006 |
| [FITC anti-human IFN-γ Antibody](https://www.biolegend.com/en-us/products/fitc-anti-human-cd45-antibody-707) | Miltenyi Biotec | 130-113-497 |
| PE anti-human TNF-α Antibody | Miltenyi Biotec | 130-118-974 |
| [Alexa Fluor® 647](https://www.biolegend.com/en-us/products/alexa-fluor-647-anti-human-mouse-granzyme-b-antibody-6067) anti-human GZMB Antibody | BioLegend | 515405 |
| PE anti-human Perforin Antibody | BioLegend | 353304 |
| PE anti-human PD1 Antibody | BioLegend | 379210 |
| PE anti-human TIGIT Antibody | BioLegend | 372704 |
| [FITC anti-human EpCAM Antibody](https://www.biolegend.com/en-us/products/fitc-anti-human-cd45-antibody-707) | BioLegend | 324204 |
| goat anti-mouse IgG FITC | Invitrogen | F-2761 |
| goat anti-mouse IgG Cy3 | Invitrogen | A10521 |
| goat anti-rabbit IgG Cy5 | Invitrogen | A10523 |
| goat anti-rabbit IgG Cy3 | Invitrogen | A10520 |
| anti-ANXA1 | Abcam | ab214486 |
| anti-TRKA | Abcam | ab302524 |
| anti-NEDD4L | Abcam | ab46521 |
| anti-GZMB | Abcam | ab208586 |
| anti-PD1 | Abcam | ab52587 |
| anti-CD3 | Abcam | ab16669 |
| anti-CD8 | Abcam | ab237709 |
| anti-Myc | Cell Signaling Technology | 13987 |
| anti-Tubulin | Cell Signaling Technology | 2148 |
| anti-PGP9.5 | Abcam | ab108986 |
| anti-HK2 | Proteintech | 66974 |
| anti-LDHA | Proteintech | 66287 |
| anti-PFKFB3 | Proteintech | 13763 |
| anti-GLUT1 | Proteintech | 66290 |
| anti-PGK1 | Proteintech | 68035 |
| anti-PDK2 | Proteintech | 15647 |
| anti-Ub | Abcam | ab134953 |
| anti-Pan-CK | Abcam | ab7753 |

**Supplementary Table3: Primer sequence**

| **Primer** | **Sequence** |
| --- | --- |
| ANXA1 | F: TGCAAGAAGGTAGAGATAAAGACAC |
|  | R: TCAGTGTTTCATCCAGGGGC |
| NGF | F: GCGCAGCGAGTTTTGGC |
|  | R: GGATGGGATGATGACCGCTT |
| PLXNB2 | F: TGGACGTCTTCGGGAAACTG |
|  | R: GTGTGCCCATCTGGGTTTCT |
| PDGFB | F: AGCGCCCATTTTTCATTCCC |
|  | R: AAGGCCCCCAAAATCGGAAA |
| CD58 | F: AGCAGCGGTCATTCAAGACA |
|  | R: GCAGCTGCTTCAAGTTACATT |
| GLUT1 | F: TGAGCATCGTGGCCATCTTT |
|  | R: AGGCATGGAACCATTCAGGG |
| GLUT2 | F: GTGCCCTGGGTACTCTTCAC |
|  | R: AACATGCCAATCATCCCGGT |
| GLUT4 | F: GCCACACTCACACAAGACCT |
|  | R: CCAGGCCTGAAATTAGCCCA |
| GLUT10 | F: CTCGCCATGGGCCACTC |
|  | R: CCAGTGCATAGTTGAGGGCA |
| PKM2 | F: TTTAGTCCCACCGAAAGGGC |
|  | R: AGATCTTGCTGCCCACTTCC |
| HK1 | F: CGCAGCTCCTGGCCTATTA |
|  | R: CTTCCACTCCGCTCGCTTTA |
| HK2 | F: GTGAATCGGAGAGGTCCCAC |
|  | R: CAAGCAGATGCGAGGCAATC |
| HK3 | F: GTGTCCTCCTGGTACGTGTG |
|  | R: TGTAGAGCGTTCCATCCACC |
| LDHA | F: CATGGCCTGTGCCATCAGTA |
|  | R: AGATATCCACTTTGCCAGAGACA |
| LDHB | F: CTGGTAGGTTTCGGCTCAGG |
|  | R: TAGGGCCTGGTTTTAGCTGC |
| PFKFB2 | F: CCAAGGCAGGGAGGGATCTT |
|  | R: TTTGCGGATCTTCATGGCCT |
| PFKFB3 | F: GATGCCCTTCAGGAAAGCCT |
|  | R: GAACACTTTTGTGGGGACGC |
| ALODC | F: AGCCTCATCTGTTTGCGGAT |
|  | R: ATGGTGACAGCTCCCTGTG |
| PDK1 | F: CTGGCTGTGGCTTCTCTAGC |
|  | R: CCGAAGTCCAGGAACTGCTT |
| PDK2 | F: TGAGCCGCTTGGATCTTTGT |
|  | R: CACCTCAGAGACGTTGCAGT |
| PDK3 | F: TGAGCGTGGCATCACAGTTA |
|  | R: CGAGGGCACAAGAGCTGTAA |
| ENO1 | F: AGGTAAACCTGCTGTGACCC |
|  | R: TAAAGCGGGACTGAACACCC |
| ENO2 | F: GTGTCTCTGGCCGTGTGTAA |
|  | R: TCTCCAGGATATTGGGGGCA |
| PGK1 | F: TGTGGTCCTGAAAGCAGCAA |
|  | R: AGTTGACTTAGGGGCTGTGC |
| PGM1 | F: GTGATCGTCCATGCAAACCC |
|  | R: CTGCATAGGTGAGGTTGGGG |
| GCK | F: CACAGTCACCTGCAGCCTAA |
|  | R: TGCCAGGATCTGCTCTACCT |
